# Proteomics as a tool to improve novel insights into skin diseases: what we know and where we should be going

**DOI:** 10.3389/fsurg.2022.1025557

**Published:** 2022-10-21

**Authors:** Sheng-yuan Zheng, Xi-min Hu, Kun Huang, Zi-han Li, Qing-ning Chen, Rong-hua Yang, Kun Xiong

**Affiliations:** ^1^ Department of Dermatology, Xiangya Hospital, Central South University, Changsha, China; ^2^ Department of Anatomy and Neurobiology, School of Basic Medical Science, Central South University, Changsha, China; ^3^ Clinical Medicine Eight-Year Program, Xiangya School of Medicine, Central South University, Changsha, China; ^4^ Department of Burn and Plastic Surgery, Guangzhou First People's Hospital, School of 173 Medicine, South China University of Technology, Guangzhou, China; ^5^ Key Laboratory of Emergency and Trauma, Ministry of Education, College of Emergency and Trauma, Hainan Medical University, Haikou, China; ^6^ Hunan Key Laboratory of Ophthalmology, Central South University, Changsha, China

**Keywords:** proteomics, skin disease, bibliometric analysis, data mining study, pathogenesis, treatment, molecular biomarker

## Abstract

**Background:**

Biochemical processes involved in complex skin diseases (skin cancers, psoriasis, and wound) can be identified by combining proteomics analysis and bioinformatics tools, which gain a next-level insight into their pathogenesis, diagnosis, and therapeutic targets.

**Methods:**

Articles were identified through a search of PubMed, Embase, and MEDLINE references dated to May 2022, to perform system data mining, and a search of the Web of Science (WoS) Core Collection was utilized to conduct a visual bibliometric analysis.

**Results:**

An increased trend line revealed that the number of publications related to proteomics utilized in skin diseases has sharply increased recent years, reaching a peak in 2021. The hottest fields focused on are skin cancer (melanoma), inflammation skin disorder (psoriasis), and skin wounds. After deduplication and title, abstract, and full-text screening, a total of 486 of the 7,822 outcomes met the inclusion/exclusion criteria for detailed data mining in the field of skin disease tooling with proteomics, with regard to skin cancer. According to the data, cell death, metabolism, skeleton, immune, and inflammation enrichment pathways are likely the major part and hotspots of proteomic analysis found in skin diseases. Also, the focuses of proteomics in skin disease are from superficial presumption to depth mechanism exploration within more comprehensive validation, from basic study to a combination or guideline for clinical applications. Furthermore, we chose skin cancer as a typical example, compared with other skin disorders. In addition to finding key pathogenic proteins and differences between diseases, proteomic analysis is also used for therapeutic evaluation or can further obtain in-depth mechanisms in the field of skin diseases.

**Conclusion:**

Proteomics has been regarded as an irreplaceable technology in the study of pathophysiological mechanism and/or therapeutic targets of skin diseases, which could provide candidate key proteins for the insight into the biological information after gene transcription. However, depth pathogenesis and potential clinical applications need further studies with stronger evidence within a wider range of skin diseases.

## Introduction

Skin, as the human body’s largest and one of the most important organs, exerts an essential function, such as protecting from external insults and maintaining microenvironment homeostasis, based on the exquisite balance of intestinal microbiota, metabolites, and so on. Once this balance is damaged or out of the compensatory range, various skin disorders occur concomitantly, resulting in physical impairment and adversely impacting the quality of life worldwide. As for this, a majority of studies have focused on the pathophysiological mechanisms of different skin diseases, benefited for accurate diagnosis and identifying the subtype of skin diseases, finding an efficient therapeutic target, and/or evaluating the treatment or nursing care effect. However, there are still multiple challenges during the basic investigation, clinical diagnosis, and treatment (e.g., a low specific mechanism or biomarker in skin diseases, preliminary inquiry of the critical molecules, ignoring the co-network in different skin diseases or other systemic changes, and arduous process in mining problem). For these aspects, the emergence of proteomics provides a systematic and reliable prediction tool or verification method (1, 2).

Mass spectrometry (MS)-based high-throughput proteomics has been regarded as a core technique for large-scale protein characterization in cells, tissues, or organisms. It mainly provides a qualitative and quantitative analysis of proteins in samples as complementary bio-information to genomics and transcriptomics, which is essential for better understanding the complex biochemical processes (3–5). From the production of proteins to function execution, it refers to various conditions for multiple post-translational mechanisms. So, proteomics has not been limited to be performed in a quantitative fashion of a conventional protein. It has also been improved to be used in the analysis of specific modifications (e.g., acetylations, methylations, and phosphorylations), leading to an insightful analysis of protein function (6–8). As we can see, the proteomic analysis aims to increase the depth of protein coverage of cells or tissues. Notably, with the development of technologies, proteomics has integrated computer elements to improve the accuracy and comprehensiveness (7–9). Meanwhile, public databases and analysis platforms began to appear and continued to innovate (10, 11), promoting proteomics data sharing and analysis applications in various fields.

In the past decade, proteomics has been used in skin diseases, which significantly contributed to skin disease's pathogenesis and clinical applications. For instance, in research on the pathophysiological mechanism, proteomics can provide candidate hub proteins (12). It can also identify similarities and differences in the proteome of different skin diseases with similar symptoms (13). Furthermore, proteomics can reveal biomarkers of different degrees of skin diseases, drug treatment targets, and treatment effects (14–16). However, the application of proteomics in skin disease research has not shown a corresponding high growth rate with the innovation of key technologies (17, 18). Moreover, only conventional proteomics methods have been used widely in most studies, and a few attempted to combine proteomics with other techniques or used multi-omics analysis (19, 20). These may make some of the limitations of proteomics irreparable, and the single application solution may also hinder the advantages of proteomics. So, the necessity to fully understand the advantages and limitations of proteomics in skin disease research is one of the keys to promoting this research. As far as we are concerned, there are no comprehensive and systemic information and suggestions for proteomics applied in skin diseases based on accurate data. Data mining and analysis is a suitable statistical and analysis method that can analyze the value and future trend of proteomics in skin diseases based on the published literature and provide more practical information in the direction of research and methods of proteomics application.

**Figure 1:**
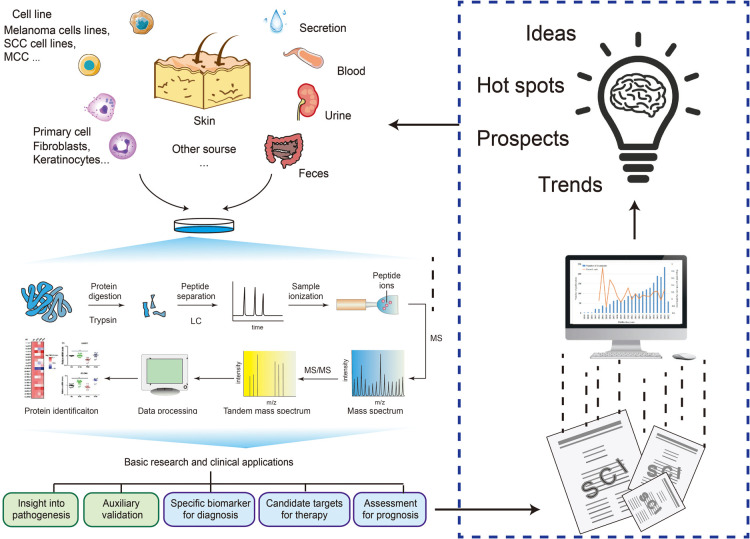
Bibliometric and data mining provide insights and trend analysis of proteomics in skin disorder. SCC, squamous cell carcinoma; MCC, Merkel cell carcinoma; LC, liquid chromatogram; MS, mass spectrum.

Generally, bibliometrics has been used as a visual analysis for trend and hot spot prediction through publications, citations, and keywords (21–23). However, since the datasets “Proteomics for Skin Disease Research” were accessed through searching online databases, the qualifier “skin disease” in the search cannot comprehensively and accurately represent all skin disease research using proteomics. Thus, we chose to use more accurate and heavier workload data mining to screen and analyze the datasets retrieved from three databases—PubMed, MEDLINE, and Embase. By manually analyzing the data, we summarized the proportion of research directions and the characteristics of time migration according to the application of proteomics in skin diseases, supplemented by quantitative analysis (Figure 1). For specific proteomics research designs, we analyzed the advantages and limitations of proteomics in dermatology based on the overall situation and specific dataset records and proposed more complete research methods. We also made a detailed record and analysis of the current research hotspots, prospects, and trends by collecting skin disease research using proteomics. The results showed that the application of proteomics in skin disease research had differences worth summarizing in terms of sample sources, techniques, and result analysis methods. In terms of pathogenesis research, we took skin cancer as a typical example and conducted a comprehensive analysis from exploring molecular functions to finding out biomarkers or take insight into the depth mechanism, which illustrated the irreplaceable importance of proteomics in skin diseases. Ultimately, we hope to summarize the contributions of proteomics and make rational research recommendations with accurate data analysis and comprehensive discussions.

## Data and methods

### Data retrieval and analysis for data mining

#### Search strategy

**Figure 2:**
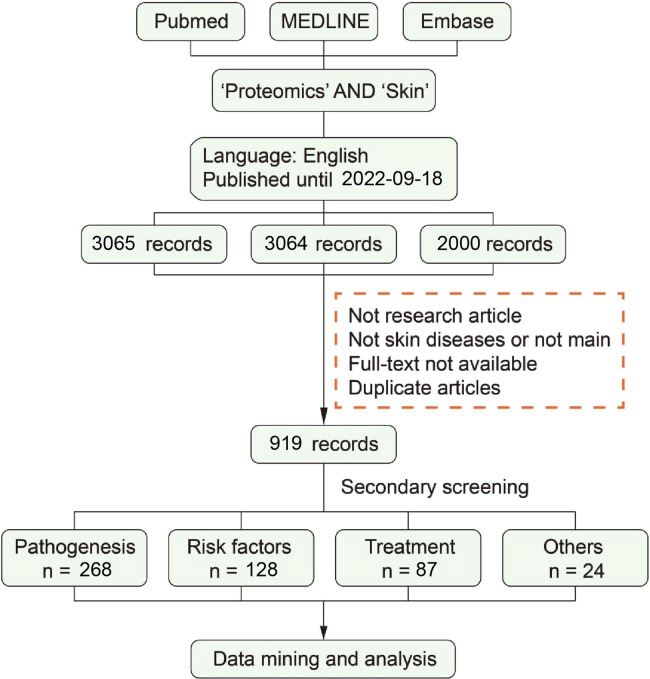
Systematic literature search and outcome identification.

We selected a systematic approach to make a document collection: PubMed, MEDLINE and Embase. The retrieval strategy of each database is customized according to the usage standard of the database and the scale of the retrieved documents. After screening within the inclusion/exclusion criteria, part of the literature has been used for a full-text screening and data collection. The process of literature screening is shown in Figure 2, and more details are as follows.

To perform a systematic analysis of skin and proteomics, we chose articles for inclusion using a data mining analysis. The terms “skin” and “proteomic” were used in the MeSH (https://www.ncbi.nlm.nih.gov/mesh) search, whereas “proteomics,” and “proteome,” “skin,” “cutaneous,” “dermal,” “dermatis” and “tegumental” were represented by other expressions.

#### Preliminary screening according to exclusion/inclusion criteria by screened titles, abstracts, and keywords

Exclusion criteria conducted were as follows:

(1) Not research article: review, letter, book, comment, etc.;
(2) Not skin diseases or not main;
(3) Full-text not available; and
(4) Duplicate articles.

After the preliminary screening was completed, 919 literature studies were included for secondary screening.

#### Secondary screening according to inclusion criteria by screened full-texts

Inclusion criteria conducted were as follows:

(1) Type of documents: original proteomic data;
(2) Topic of documents: studied on skin diseases using proteomics; and
(3) Contents of documents: Reporting of at least one statistically significant protein were included;

After the secondary screening was completed, 486 literature studies were included for secondary screening.

#### Grouping the literature in tables

Literature studies included finally were divided into several groups according to research topics and the four groups are as follows: (1) Pathogenesis; (2) Risk factor; (3) Treatment; (4) Others.

#### Data extraction and analysis

Then, we made whole summaries of the literature in publication, situation of development, and analyzed the details in each table. In the process of selecting article data, we mainly extracted disease types, proteomic sample sources, key enrichment pathways, and in-depth research based on differential proteins. In addition, we extracted risk factors for disease in the Risk factor group, and considering that some studies have also given novel solutions for the corresponding factors, we also extracted Options for risk factors in particular. For the Treatment group, we have additionally extracted treatments for better understanding. Comprehensively analyzing these contents, we have drawn some research characteristics of proteomics in various aspects.

### Data retrieval and analysis for bibliometrics study

To make our summary more credible, we also used bibliometrics to analyze the characteristics of published articles from the perspective of big data. Due to “skin” or “skin diseases” being inaccurate in searching the literature from the databases, we chose skin diseases using data mining. The search was conducted in the Web of Science (WoS) Core Collection database with citation indexes, including Science Citation Index Expanded (SCIE), Social Science Citation Index (SSCI), and Emerging Sources Citation Index (ESCI). A total of 2,060 documents were retrieved to make visual bibliometric analysis with the duration from 1 Jan 2002 to 18 Sep 2022.

## Results

### The overview of the application and distribution of proteomics in skin diseases

**Figure 3:**
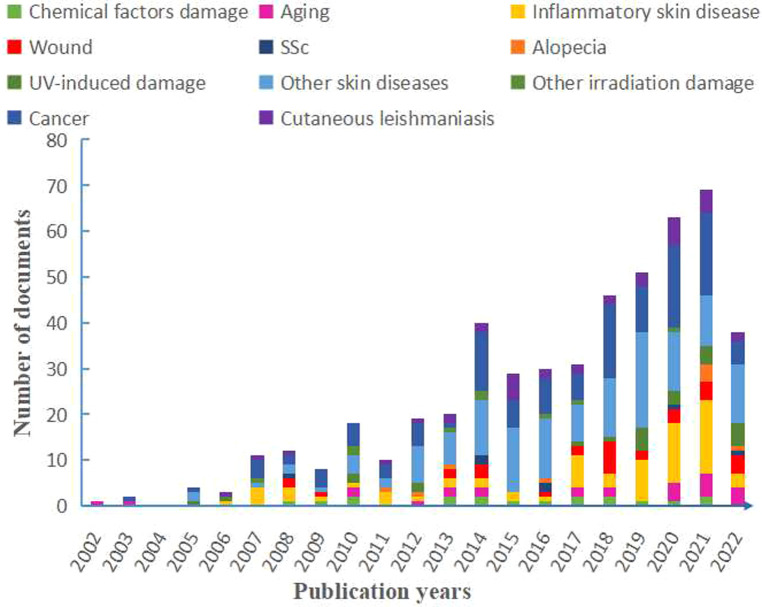
Distribution of publications on the studies of skin diseases according to year and each part contributions have also shown. SSc, systemic sclerosis; UV, ultraviolet; AD, atopic dermatitis.

All included articles were counted according to respectively related diseases. According to the type of skin diseases, we classified the diseases in different color and performed the publication search by year through manual statistics (Figure 3). From 2002 to 2021, the total number of publications was on an upward trend with volatility. Especially from 2015 to 2021, a successive growth trend in proteomics was shown using the field of skin, and our bibliometric data also indicated this increasing trend (Supplementary Figure S1). Among these skin diseases, skin cancer seems to be a major part of studies using proteomics that reached the highest growth rate in 2018 and peaked in 2020 and 2021. Also, the publications on psoriasis and atopic dermatitis (AD) showed an increased trend and reached a peak in 2021. As for skin wounds, the growth trend of publications leveled off owing to a massive growth in 2018. The overall trend of cutaneous leishmaniasis has been relatively stable from 2013 and increased slightly in the past two years (Figure 3). In addition, our bibliometric data have showed that some keywords of skin disease that have co-occurred in recent years are consistent with the above results (Supplementary Figure S2).

**Figure 4:**
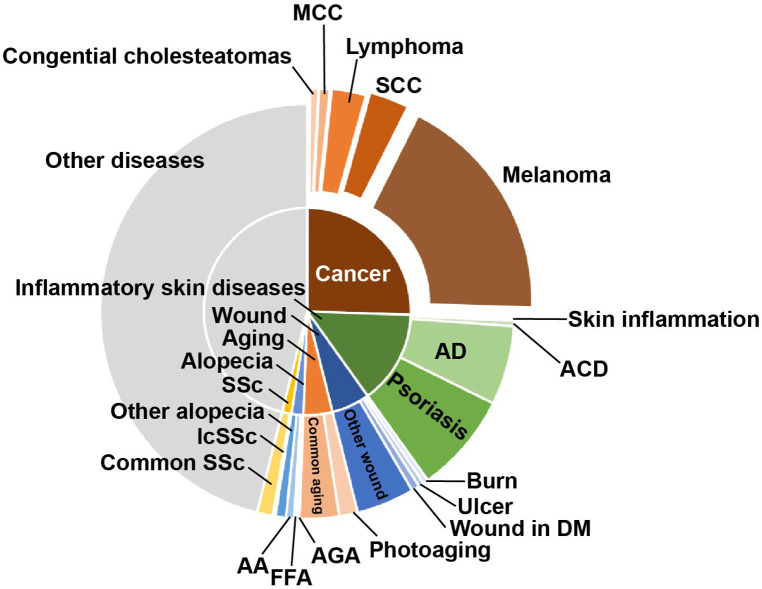
The proportion of research on the different skin diseases. The main application directions of proteomics are skin cancer and inflammatory skin diseases, among which melanoma, AD, and psoriasis are the main ones. SCC, squamous cell carcinoma; MCC, Merkel cell carcinoma; AD, atopic dermatitis; ACD, atopic contact dermatitis; DM, diabetes mellitus; FFA, frontal fibrosing alopecia; AA, alopecia areata; AGA, androgenetic alopecia; SSc, systemic sclerosis; lcSSc, limited cutaneous SSc.

In addition, to further analyze the research on skin diseases using proteomics, we noted the diseases with the above conditions. The proportion of different skin diseases varied widely across all studies. Here, we mainly highlighted cancer, inflammatory skin diseases, wound, aging, alopecia, and systemic sclerosis (SSc) (Figure 4). Cancer accounted for the largest proportion of all disease categories (25.514%). Among these, melanoma (18.107%) had the highest proportion, followed by squamous cell carcinoma (SCC) (3.086%). In addition, there were studies involving lymphoma, Merkel cell carcinoma (MCC), and cholesteatoma. Inflammatory skin disease is the second largest category accounting for 14.609%. Psoriasis and AD are the primarily researched diseases, accounting for 7.819% and 6.173%, respectively. Wound, aging, alopecia, and SSc account for a smaller proportion, whose subordinate diseases are more diverse. Cutaneous leishmaniasis, UV-induced damage, and chemical factors damage were classified as other skin diseases here because they are not further subdivided.

Overall, the increased trend line and broad application showed good progress in the study of skin diseases using proteomics. The range of skin diseases is large and diverse within complex pathogenesis, and proteomic analysis provides a systematic analysis of the proteins during the disease development. Notably, proteomic analysis has begun to be incorporated in many other diseases in recent years (Figures 3, 4). However, the sample source, research significance, and their depth and breadth are different, so we conducted full-text mining to obtain more detailed information.

### Pathophysiological research analysis

#### Proteomics in pathogenesis, taking skin cancers as the example

A total of 268 articles studied on pathophysiology were obtained and divided into seven parts of different types of skin diseases, including skin cancer, inflammation skin disease, wound, skin aging, hair disorder, systemic sclerosis, and others. According to the data, we suppose that there is something that is common to these skin diseases. For a better understanding of the proteomic application in skin disease, we used skin cancer, the most significant proportion in the above profiling, as a typical example in pathogenesis compared with other skin diseases. To elucidate some of the crucial mechanisms of skin cancer and the role of proteomics, we screened the differential proteins in each article, shown as the top upregulated and downregulated ones in Table 1 and Supplementary Table S1.

**Table 1:** Mechanism of squamous cell carcinoma pathogenesis within proteomic analysis.

Type of disease	Sample	TOP differential proteins	Biological process of cancer	Highlighting mechanism	Depth mechanism	Ref.
SCC	SCC cell lines	ANO1, CE350, NRBF2, NSUN4, K1C14, KT33B, HORN in 10% O_2_; TRI18, TRY3, ZN714, VAT1, AL1B1 in 5% O_2_; TRFL, HBA, PI3R4, CTL1, K2C8, TTLL1, FGFR2, DKK2, CCD73, STON2, SHRM3 in 1% O_2_	Levels of hypoxia	Energy, metabolism, nucleic acid regulation	—	Głuszko et al. (2021)
SCC	SCC cell lines	Up: CK19, tropomyosin 1α chain, HSP27, CK8, AHCY, NNMT, GSTO1	Proliferation, invasion (NNMT)	—	NNMT knockdown inhibits cell migration and invasion	Hah et al. (2019)
SCC	SCC cell lines	A431: FUCA2, LRG1, MAGEA4, CSF1, PROS1, PLAT, LCP1, AXL, EGFR, DNAJB11;	—	Metabolism, mTOR, PI3K-AKT, RAP1 signaling	Secretome of A431 cancer cells accelerates migration and proliferation	Hoesl et al. (2020)
		HaCaT: SERPINB7, MMP13, GSN, DSG4, MXRA5, SEMA3A, FN1, SPARC, MMP2, DSC3				
SCC	SCC cell lines	Down: AKT2, MAP4K4, PRKCD, ERBB2, CLK2, STK10, MAP2K1, EGFR, PTK2B, MAP2K2;	Invasion	MAPK, mTOR pathways; adhesions, skeleton	YAP1 and SOX2 are related to mesenchymal and epithelial transcriptional programs	Pastushenko et al. (2021)
		Up: YES1, CDK11, PRPF4B, BMP2K, CDK12, PNKP, MNAT, MINK				
SCC	SCC cell lines	Up: FABP5, S100A9, S100A8, SPRR1A, AKR1C3, AKR1C2, SPRR1B	Proliferation	—	—	Shintani et al. (2021)
SCC	SCC cell lines	CDK1^T14,Y15^, EIF4EBP1^T46,T50^, EIF4B^S422^, AKT1S1^T246,S247^, CTTN1^T401,S405,Y421^, CAP1^S307/309^ in K8-S73A/D mutant; CTTN1^T401,S405,Y421^, BUB1B^S1043^, CARHSP1^S30,S32^ in K8-S431A/D mutant	Metastasis, Proliferation, Invasion	Junction, JNK/SAPK, CDC42 signaling, Hippo signaling	—	Tiwari et al. (2017)
SCC	SCC cell lines	—	—	Cholesterol and steroid metabolic process	HMGCS1 partly was related to A431 cell proliferation	Xu et al. (2022)
SCC	SCC cell lines	KRT8, KRT14, KRT18	—	—	—	Yamashiro et al. (2010)
SCC	SCC cell lines	—	—	—	—	Yanagi et al. (2018)
SCC	Human [SCC (*n* = 5)]	Up: G3BP, Filamin-A, NDRG1, Myosin-9, Plectin, 40S Ribosomal protein S4, Actin-related protein 2/3 complex subunit 1B, Fascin, Transgelin, Superoxide dismutase [Mn];	—	Cell survival, proliferation	—	Azimi et al. (2016)
		Down: FLG2, Suprabasin, FLG, Dermokine, Apolipoprotein EArginase-1, Galectin-7, Desmoglein-1, Desmoplakin, Collagen alpha-1(III) chain, Corneodesmosin				
SCC	Human [SCC (*n* = 20)]	—	—	Gene, metabolism	—	Chen et al. (2021)
SCC	Human [samples of primary (*n* = 19 patients, *n* = 20 lesions) and metastatic SCC lesions (*n* = 20 patients, 25 lesions)]	Up: ISG15 ubiquitin like modifier, APOA1, MARCKS, EFHD2, STMN1, ACBD3;	Metastasis	Invasion, migration, immune response	—	Azimi et al. (2020)
		Down: CMA1, CST6, KRT79, CPA3, APCS, DMKN				
SCC	Human [primary (*n* = 52), metastasize (*n* = 53)]	Up: TNC, POSTN, TGFB1, PRDX5, SFPQ, FGB, LCP1, PHB2, HNRNPA2B1, P4HB;	Metastasis	PI3K-Akt signaling	—	Shapanis et al. (2021)
		Down: CALML5, KRT2, KRT6B				
SCC	Human [AK (*n* = 19), SCC (*n* = 21), HCs (*n* = 40)]	—	—	MEK-ERK, EGFR, mTOR pathways	—	Einspahr et al. (2021)
SCC	Human	—	—	Cell motility, cell death, survival, growth, proliferation, morphology	KRT17 protein is related to caspase-mediated degradation, supporting by altered TMS1-NF-κB signaling	Tiwari et al. (2018)

SCC: squamous cell carcinoma; HCs, human controls; AK, actinic keratosis; MAPK, mitogen-activated protein kinase; PI3K, phosphatidylinositol-4,5-bisphosphate 3-kinase; mTOR, mechanistic target of rapamycin; JNK/SAPK, c-Jun N-terminal kinase/stress-activated protein kinase; CDC42, cell division cycle 42; RAP1, role of ras-associated protein 1; EGFR, epidermal growth factor receptor; Panx1, pannexin 1; AKT, protein kinase B; NNMT, N-nicotinamide methyltransferase; YAP1, yes-associated protein 1; SOX2, SRY-box transcription factor 2; HMGCS1, 3-hydroxy-3-methylglutaryly-CoA synthase 1; NF-κB, nuclear factor-kappaB; KRT17, keratin 17; AKT2, AKT serine/threonine kinase 2; CLK2, cdc2-like kinase 2; MAP2K1, mitogen-activated protein kinase kinase 1; MAP4K4, mitogen-activated protein kinase kinase kinase kinase 4; PRKCD, protein kinase C, delta; ERBB2, epidermal growth factor receptor 2; STK10, serine threonine kinase 10; PTK2B, protein tyrosine kinase 2 beta; MAP2K2, mitogen-activated protein Kinase Kinase 2; YES1, YES proto-oncogene 1 tyrosine kinase; CDK11, cyclin-dependent kinase 11; PRPF4B, PRP4 pre-mRNA processing factor 4 homolog B; BMP2K, BMP-2-inducible protein kinase isoform b; PNKP, polynucleotide kinase 3'-phosphatase; FABP5, fatty acid-binding protein 5; S100A9, S100 calcium-binding protein A9; AKR1C3, aldo-keto reductase family 1 member C3; SPRR1B, small proline-rich protein 1B; TNC, tenascin C; TGFB1, transforming growth factor, beta 1; PRDX5, peroxiredoxin 5; LCP1, lymphocyte cytosolic protein 1; PHB2, prohibitin 2; P4HB, prolyl 4-hydroxylase beta-subunit; CALML5, calmodulin-like 5; KRT, keratin; NRBF2, nuclear receptor binding factor 2; CTL1, choline transporter-like 1; K2C8, keratin, type II cytoskeletal 8; TTL1, Tubulin tyrosine ligase-like family, member 1; FGFR2, fibroblast growth factor receptor 2; DDK2, Dickkopf-2; STON2, Stonin 2; LRG1, leucine-rich alpha-2-glycoprotein 1; CSF1, colony-stimulating factor 1; PROS1, protein S; PLAT, plasminogen activator; MMP13, matrix metallopeptidase-13; GSN, gelsolin; DSG4, desmoglein 4; MXRA5, matrix-remodeling associated 5; SEMA3A, semaphorin 3A; FN1, fibronectin 1; SPARC, secreted protein acidic and rich in cysteine; DSC3, desmocollin 3; CK19, cytokeratin 19; AHCY, adenosylhomosysteinase; GSTO1, glutathione S-transferase omega 1; EIF4EBP1, eukaryotic translation initiation factor 4E binding protein 1; EIF4B, eukaryotic translation initiation factor 4B; AKT1S1, AKT1 substrate 1; CAP1, cyclase-associated protein 1; CARHSP1, calcium-regulated heat stable protein 1; G3BP, galectin-3-binding protein; FLNA, Filamin-A; NDRG1, N-myc downstream regulated gene 1; FLG2, Filaggrin-2.

In data, skin cancers mainly included melanoma, SCC, lymphoma, etc. Some differential proteins with similar functions in cancer appeared in different studies. Moreover, these proteins can be mainly clustered into inflammation, immunity, survival/death, metabolism, metastasis, cyclin-dependent kinase (CDK), and heat shock protein (HSP). (Many of the following proteins have multiple functions, and the clustering here is only based on the main function, rather than an absolute distinction.)

Inflammation-related proteins are detected to upregulate in nearly all skin cancers, such as the interleukin family in melanoma, the S100 calcium-binding proteins in SCC, and tumor necrosis factor(TNF) in cutaneous T-cell lymphomas (CTCL). Some immunity-related proteins were detected in melanoma, like CD14 as a particular protein in stage II melanoma and upregulated Endoglin/CD105. The proteins related to the survival of melanoma cells mainly include Galectin-3, Survivin, Dickkopf (DKK), Bcl-2, and so on.

Proteins related to the metabolism of melanoma cancer cells mainly include heme oxygenase-1 (HO-1/HMOX1), Cathepsin S, Progranulin, FBXO32, etc. For SCC, they mainly include CDC-like kinase 2 (CLK2), serine/threonine kinase 10 (STK10), aldo-keto reductase family 1 member C3 (AKR1C3), etc. There are also some proteins related to cancer migration, such as intercellular adhesion molecule 1 (ICAM-1), tubulin and vascular cellular adhesion molecule-1 (VCAM-1) in melanoma, transforming growth factor-*β* (TGF*β*), and calmodulin-like 5 (CALML5) in SCC. In addition, some proteins are involved in many life activities such as tumor cell proliferation and migration, such as serine/threonine kinase 2 (AKT2).

In addition to functional distinction, some proteins can also be considered promising markers for skin cancer diagnosis. HSPs are associated with many characteristics of cancer, such as cell proliferation and metastasis. In addition, there are Survivin, CDK12, Vimentin, and Galectin-3. These proteins may be potential biomarkers for some skin cancers. In addition, some studies focus on markers in the pathogenesis process, but it has not been confirmed experimentally (Table 1, Supplementary Table S1).

Based on differential proteins, many studies have further enriched their functions to explain critical pathways and biological processes in the development or progression of skin cancer. In melanoma-related proteomic studies, PI3K/AKT pathway, mTOR pathway, and mitogen-activated protein kinase (MAPK)/ERK pathway are the most frequently mentioned signaling pathways (Supplementary Table S1). Upstream of the mTOR pathway, PI3K/AKT signaling initiates several selective signaling cascades that lead to increased cell growth and proliferation (24). Whether it is tripartite motif-containing 14 (TRIM14)-promoted melanoma progression or Hey1-promoted melanoma invasion and migration, the PI3K/AKT pathway is an important part (25, 26). Activation of the mTOR signaling pathway plays a role in melanocytic tumorigenesis by regulating extracellular signals that control cell growth, proliferation, and apoptosis (27, 28). MAPK/ERK pathway also plays a vital role in the tumorigenic effects of transcription co-activators 1–3 (29). In addition, most enrichment results focused on biological processes, especially functions related to tumor progressions such as cell adhesion, proliferation, and migration. SCC is similar to melanoma, except that JNK/SAPK signaling, cell division cycle 42 (CDC42) signaling, and Hippo signaling also appears in the pathway enrichment of the former (Table 1, Supplementary Table S1).

From the data and analysis of skin cancer, proteomics can provide changes in fundamental proteins for disease research and can also explore important functional changes in the pathogenesis through enrichment or in-depth research. This situation is also prominent in other skin diseases. Whether searching for psoriasis biomarkers or the research on the pathogenesis of AD, the application and analysis of proteomics is similar to skin cancer.

#### Sample of proteomics

The sources of samples for proteomic analysis were diverse, and the mainstream sample used for each disease was different. The choices of samples are related to the characteristics of the disease and the purpose of the proteome.

The most important source of samples for cancer was tumor cell lines cultured *in vitro*, accounting for 59.302% (Table 1, Supplementary Table S1). The second was cancer tissue samples, most of which were cancer samples of the skin, and a small number of studies used fibroblasts (30), serum (31), and metastatic cancer tissue (32) to study the progression and metastasis of cancer. Compared with skin cancer, blood samples from diseased individuals are essential and frequently used in research on inflammatory skin diseases (41.818% in Table 2 and Supplementary Table S2). Blood samples include serum, plasma, and biologically active substances and cells in the blood environment. These samples provide an accurate and detailed reflection of inflammatory changes in skin diseases. Based on these changes, biomarkers can obtain biomarkers (33), diseases can be distinguished (13, 34), and also the pathogenesis can be studied with hub proteins (35, 36). In addition, the use of skin tissue samples and primary cell samples was also common. In recent years, studies have begun to use blood samples and skin tissue samples from the same individual to obtain more comprehensive information on pathological changes (37, 38).

**Table 2:** Mechanism of atopic dermatitis pathogenesis within proteomic analysis.

Type of disease	Sample	Highlighting mechanism	Depth mechanism	Ref.
AD	Human [AD (*n* = 12), HCs (*n* = 13)]	Localization, regulation of biological quality, platelet activation, etc.	—	Chang et al. (2021)
AD	Human [AA (*n* = 35), HCs (*n* = 36), psoriasis (*n* = 19), AD (*n* = 49)]	Atherosclerosis signaling, immune pathways, cardiovascular pathway	—	Glickman et al. (2021)
AD	Human [AD (*n* = 4), HCs (*n* = 7), spontaneously healed AD (*n* = 4)]	—	—	Rindler et al. (2021)
AD	Human [AD (*n* = 34), HCs (*n* = 20)]	—	Insufficiency of IL-37 leads to dysregulation of serum protein and skin disruption in AD	Hou et al. (2021)
AD	Human [AD (*n* = 8), HCs (*n* = 8)]	Biological regulation, cellular component organization, etc.	—	Morelli et al. (2021)
AD	Human [AD (*n* = 20), HCs (*n* = 28)]	—	—	Pavel et al. (2020)
AD	Skin suction blisters and skin	—	—	Rojahn et al. (2020)
AD	Human [18–40 years old (*n* = 26), 41–60 years (*n* = 24),>60 years (*n* = 21), HCs (*n* = 37)]	Th1/Th2 differentiation, IL-4-mediated signaling, IL-5-mediated signaling, etc.	—	He et al. (2020)
AD	Human [AD + FA (*n* = 21), AD (*n* = 19), HCs (*n* = 22)]	Inflammatory response, glycolysis, oxidative stress response	—	Goleva et al. (2020)
AD	Human [AD (*n* = 20), HCs (*n* = 7)]	—	—	Umayahara et al. (2020)
ACD	Mice TGs	Response to stimulus, metabolic process, immune system process, etc.	—	Su et al. (2020)
AD	Human [AD (*n* = 76), HCs (*n* = 39)]	—	—	Leonard et al. (2020)
AD	Human	—	—	Yin et al. (2019)
AD	Human [AD pediatric (*n* = 30), healthy pediatric (*n* = 19), AD adult (*n* = 58), healthy adult (*n* = 18)]	—	—	Brunner et al. (2019)
AD	Human [HCs (*n* = 84),severe AD (*n* = 50), moderate AD (*n* = 123)]	—	—	Mikus et al. (2019)
AD	Human [HC (*n* = 10), AD (*n* = 20), CD (*n* = 10), AD and CD (*n* = 10), psoriasis (*n* = 12)]	—	—	Wang et al. (2017)
AD	Human [psoriasis (*n* = 22), AD (*n* = 59), HCs (*n* = 18)]	—	—	Brunner et al. (2017)
ACD	Dendritic-like cell line	Cell signaling, transcriptional regulation, protein transport, ubiquitination, etc.	MLK is one of FITC targets leading the specific protein haptenation and the subsequent pathway of dermal dendritic cells activation	Guedes et al. (2017)
AD	Spleen and thymus Treg cells	—	—	Lee et al. (2016)
AD	AD-NC/Nga mice	—	—	Kawasaki et al. (2014)
AD	Human [EH− (*n* = 18), EH+ (*n* = 17), non-atopic controls (*n* = 6)]	Skin barrier, generation of natural moisturizing factor	—	Broccardo et al. (2011)
AD	Human [AD (*n* = 8), HCs]	—	—	Kim et al. (2008)
AD	Human [ADe (*n* = 14), ADi (*n* = 10), HCs (*n* = 14)]	—	—	Park et al. (2007)
AD	Human [ADe (*n* = 14), ADi (*n* = 10), HCs (*n* = 14)]	—	—	Park et al. (2007)
AD	Human [ADe, ADi, HCs]	—	—	Park et al. (2006)
AD	Human [ADe, ADi, HCs]	—	—	Park et al. (2004)

AD, atopic dermatitis; ACD, atopic contact dermatitis; HCs, human controls; AA, alopecia areata; TGs, trigeminal ganglions; CD, contact dermatitis; EH, eczema herpeticum; ADe, extrinsic AD; ADi, intrinsic AD; PsA, psoriatic arthritis; FITC, fluorescein isothiocyanate; MLK, mixed-lineage protein kinase.

In the study of other diseases, there were also applications of specific samples corresponding to particular diseases (Supplementary Tables S5–S8). As for skin wounds, we often focused on the surrounding skin tissue but ignored the exudates of the wound. Studies by Krisp et al*.*, van der Plas et al*.,* and Bekeschus et al*.* reported that the exudates of skin wounds might have important implications for the progression of wounds (39–41). In the study of bullous pemphigoid, blister fluid was used as a sample for proteomic analysis (42). Moreover, with the continuous improvement of online databases in recent years, some studies have begun to download online data for analysis (43, 44). However, the skin proteomic database still needs to enrich and improved. The use of online analysis may be one of the future development trends of skin disease research.

#### Application of proteomics in skin pathophysiological research

Turning back to the total articles of skin disease benefited for proteomics, most of them were based on proteomic analysis to raise a presumption and explore the pathology of skin diseases. The typical analysis process consists of five steps: sample acquisition, protein purification, qualitative and quantitative, acquisition of differential protein information, and validation (Western blot, ELISA, etc.). However, this routine process can only provide insights into the study of the pathological mechanism but cannot concretize the results of protein profiling (45).

With the improvement and promotion of bioinformatic platforms, such as the Kyoto Encyclopedia of Genes and Genomes (KEGG), Gene Ontology (GO), and Ingenuity Pathway Analysis (IPA), the results of proteomics can be analyzed online to correlate with information on cellular pathways and biological processes. The proteomic protein function analysis could provide comprehensive and specific information for the progress of pathogenesis. According to the statistical results of the pathophysiological group, 50.988% of the studies conducted functional analyses of the online database on the proteome results (Tables 1, 2, Supplementary Tables S1, S2, S5–S8). Taking the melanoma study by *Konstantakou* et al. as an example, after obtaining the proteome, a series of online database analyses were used to provide insights into the core proteins and pathways from tumorigenesis to therapeutic targets (2).

In addition, some studies use proteomics as a reliable method for validating research findings (46) or replenishing proof of results (47). These may be new approaches to applying proteomics to skin disease research. Online database analysis of proteomes is not a necessary research process. Follow-up studies based on proteomic analysis results can be comprehensive (such as GO analysis) or precise (only targeting one core protein or core pathway). For example, PSMB9 in systemic lupus erythematosus (SLE) and TRIM29 in SCC were obtained through proteomic screening, and further functional studies were conducted precisely (12, 48). Moreover, a study identified the critical mechanism associated with melanoma metastasis through specific changes obtained by proteomics (49). At present, such precise functional research has a more specific role in promoting the research on the pathophysiological mechanism of skin diseases. However, as the basis of precise studies, extensive functional studies like GO analysis and KEGG analysis are essential and non-negligible.

### Risk factors research analysis

A total of 128 articles were included in the risk factor group, and these articles were divided into two tables according to biological risk factors and abiotic risk factors. Non-biological risk factors include UV exposure, chemical exposure, and other irradiation, while biological risk factors are all about microbial skin infections (Supplementary Tables S3, S9). Among the 44 articles related to non-biological risk factors, the research on radiation (including UV and laser) accounts for the most significant proportion (50.000%). As for biological risk factors, *Leishmania* and its subspecies infection studies account for the vast majority (46.913%).

The focus of research on risk factors is to lead a guideline for preventing or treating diseases, which is further beneficial for discovering the critical pathogenic proteins and mechanisms of risk factors.

### Treatment research analysis

A total of 87 articles were included in the skin disease therapy. Skin cancer has the highest number of studies, while the wound is as the second but less than half the number of cancers. The number of remaining studies on psoriasis, aging, UV-induced damage, and AD is not much different (Supplementary Table S4).

Among 28 studies related to skin cancer therapy, 19 studies used *in vitro* cells, and 89.473% were tumor cell lines. For samples, de Groot et al. have reported that combined treatment of IACS-010759 (IACS) and atorvastatin (STN) could induce tumor regression, and they further explored the mechanism MAPK pathway using functional proteomic analysis (50). In researching glucocorticoid treatment for vitiligo, Qian et al. pioneered urine as a sample source for proteomics to identify the differentially expressed proteins and ingenuity pathway analysis (such as retinol binding protein-1 and torsin 1A interacting protein 1) (51). In these studies, proteomics exerts as a screener, accurately reflecting changes in proteins before and after interventions. Similar to the study of pathophysiological mechanisms, only a few studies carried out precise therapeutic mechanism studies based on core proteins after obtaining the proteomic results. However, unlike the study of pathophysiological mechanisms, the application of proteomics in therapeutic research was more inclined to investigate the effectiveness of the intervention substance or the main therapeutic effect of the intervention substance. For example, BRAF inhibitors were used in melanoma therapy, and MS-based chemical proteomics were used to identify never-in-mitosis-gene-A related kinase 9 (NEK9) and CDK16 as unique targets of dabrafenib (52). The situation may be related to the different purposes of the two types of research. The study of pathophysiological mechanisms explores the possibility of disease grading and treatment, and the study of therapy is to judge the degree of cure and effectiveness.

## Discussion

**Figure 5:**
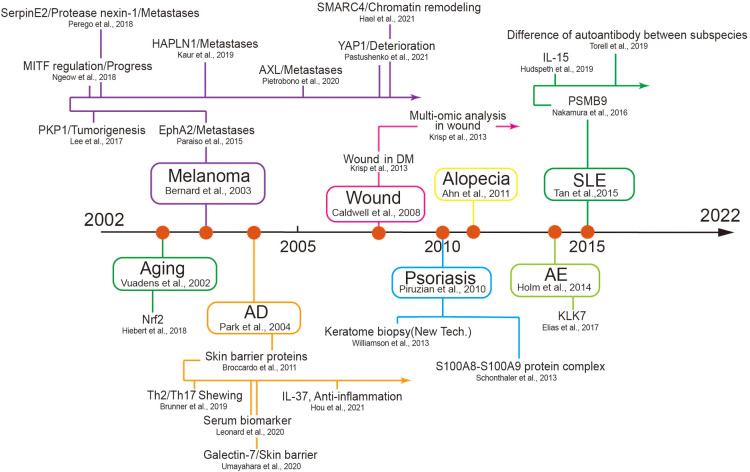
The timeline of some key discoveries in the field of the skin disease using proteomics. The first records of proteomics used in several diseases and their subsequent development are listed here. We selected primary skin diseases that began in different periods to list and used high-impact factor articles as developing nodes to display in the follow-up development process AD, atopic dermatitis; SLE, systemic lupus erythematosus; AE, atopic eczema; PKP1, plakophilin-1; HAPLN1, hyaluronan and proteoglycan link protein 1; AXL, AXL receptor tyrosine kinase; YAP1, yes-associated protein 1; MITF, microphthalmia-associated transcription factor: DM, diabetes mellitus; KLK7, kallikrein-7.

Over the hundreds of years of studies in skin diseases, the curve of its annual publications showed a sharply increasing trend in recent 20 years. During the preceding 12 years covered by the proteomic analysis utilized in the studies of skin diseases, a number of annual publications have contributed to the development of skin diseases, which reached a peak in 2021. Among these, skin cancer as a major part exerted a sharply increasing trend in recent five years for the cancer hotspot or most research groups, a wide range of samples and multiple cell lines, and high prevalence, a limited therapeutic effect, and sorely needed treatment targets (53). Skin inflammation disease, as a major category of skin disease that ranked second, may be related to its prevalence with a well therapeutic response (54). We have summarized the timeline of some key discoveries in different skin diseases according to our data (Figure 5). In all, this curve suggests that an increasing number of researchers pay attention to improved technologies, which indicates a continuously increasing hotspot of proteomic analysis in skin disorders over the next few years.

Proteomic assays have been extensively used in the field of skin, providing significance to nucleotide sequences by giving a direct link to biological activity, such as functional proteomics and phosphoproteomics (55, 56), so that the knowledge of structural–functional cellular complexities and their changes under skin physiological and pathological conditions could advance at a faster pace (57). Numerous studies have identified skin lesion-related changes (including systemic changes) in protein expression or modification to enrich the specific pathogenesis well or find a special biomarker for diagnosis and a therapeutic target for a particular disease (58). Recently, a more improved and delicate proteomic analysis has been developed, such as spatial proteomics (protein subcellular localization tightly combined with its functions) (59), multi-omics analysis (genetics-proteomics) (60), and single-cell proteomics (61).

As for the pathogenesis of skin diseases, we analyzed the application and characteristics of proteomics in skin disease and selected skin cancer as a typical example for further explanations. We enumerated differential proteins with similar functions. Whether related to inflammation or cancer cell survival, these proteins are undoubtedly of great significance for the study of skin cancer. S100A9 is a protein involved in the recruitment of immune cells and has been shown to be a molecule in the epidermal layer of the skin that controls skin tumor formation (62). As survival-related molecules, DKK proteins can also affect the phenotype of melanoma in various ways (63, 64), and studies have also shown that the invasive activity of melanoma cells can be inhibited by DKK1 (65). Knockdown of FBXO32, which is related to the metabolism of melanoma cancer cells, was able to induce global changes in melanoma gene expression profiles and was shown to correlate with melanoma cell migration, proliferation, and tumor development (66). ICAM-1, a cell surface glycoprotein and adhesion receptor, was shown to be expressed together with LFA-1 under melanoma-endothelial cell co-culture conditions to facilitate melanoma metastasis *in vitro* (67, 68). However, there are still many high-fold change proteins whose real functions are still unclear (Table 1, Supplementary Table S1). In addition, we also tried to find some common biomarkers, such as HSPs, Survivin, Galectin-3, and other markers that have been experimentally verified. The detection of HSPs in the serum of sick individuals can play an essential role in cancer diagnosis (69). Survivin, one of the inhibitors of the apoptosis protein family, is overexpressed in cancer and has been proved to be a biomarker for cancer diagnosis (70). CDK12, a member of the CDK family, has also been shown to play an important role in various human cancers, suggesting that it is a potential biomarker of cancer (71). Vimentin is proved to be not only the diagnostic marker but also the hematogenous metastasis predictor for melanomas clinically (72). Galectin-3 shows high serum levels in advanced melanoma patients and is also regarded as an important biomarker for prognosis in stage III and IV melanoma patients (73). There are also many potential biomarkers that have not been further confirmed. For instance, the study by Welinder et al*.* identified several proteins correlated with tumor tissue content by combining proteomics with histopathology and clinical outcomes, of which HEXB, PKM, and GPNMB were relatively prominent and may serve as biomarkers in progression from stage III to IV melanoma (74).

Proteomics is mainly involved in screening differential proteins and enriched functions, as well as in the search for biomarkers in the pathogenesis of skin cancer. Most of the research relies on proteomics to obtain the general protein set related to cancer, obtain the enriched protein function through platform analysis or other approaches, and then highlight or research the part related to cancer. For example, Eumorphia et al. used nano-liquid chromatography-tandem mass spectrometry (nLC-MS/MS) proteomic technology to dissect the deep proteome of WM-266-4 human metastatic melanoma cells and found 6,681 unique proteins. Ultimately, they focused on 1433G and ADT3 proteins, which are related to epithelial-to-mesenchymal (EMT) and mesenchymal-to-epithelial (MET) programs (2). Some studies focused on specific proteins and explored the relationship between proteins and cancer. For example, to explore the role of heparin-binding protein 17 (HBp17) and FAT1 in the progression of cutaneous squamous cell carcinoma, both adopted the knockout-first pattern and finally proved their mechanisms in promoting keratinization and deterioration, respectively (75, 76). Identifying FAM83H as a TRIM29 interactor through comprehensive proteomic and immunoprecipitation analysis is similar (48).

In exploring biomarkers, researchers obtain candidate proteins by comparing differential proteins from the tissue or cells from different stages of diseases. However, the screened candidate proteins often cannot become efficient markers. Whether for tumors and tumors, or tumors and other diseases, due to the existence of similar biological processes, the specificity of differential molecules expressed in clusters is not enough to distinguish similar diseases. At present, the solution is to adopt the combined detection of immunolabeling to meet the specific identification, but this is not a universal and cost-effective method. In some respects, proteomics has similar functions to genomics, transcriptomics, etc. However, in particular, the proteome is dynamic, regulated by post-translational modifications (PTMs), and the prediction of impaired PTMs cannot be achieved by genomic and transcriptomic analysis but only by proteomic approaches (77). Therefore, developing proteomic technologies and applications is very important for skin cancer. Likewise, these conditions are suitable for other skin disorders.

Among the research on skin disorders, proteomics seems to be a key part and a research hotspot in skin research. According to our data, we have found that proteomics contributed to a more profound discovery of the physiology and pathology of the skin. Both in circulation and skin of systemic sclerosis, CXC-l4 was found as a special biomarker *via* proteome-wide analysis, which provided a meaningful guideline for clinical diagnosis both for the presence and progression of complications (78). As for shrimp allergen analysis (most of the allergens were protein), Pen m2 was identified as a novel cross-reactive crustacea allergen through matrix-assisted laser desorption ionization time-of-flight mass spectrometry analysis (79). For depth mechanism, Yoon et al*.* found a specific defect in an internal ribosome entry site-dependent translation in X-linked dyskeratosis congenita using an unbiased proteomic strategy (80). Also, secretory proteome has been used for skin-related microbiomes, such as to reveal the pathogenicity of extracellular hydrolases from dandruff-associated *Malassezia* (81). Proteomics has been used to identify protein-interacting proteins, such as ERK-interacting protein analysis during cell fate decisions (82). Besides research articles, various high-cited reviews have also pointed out that proteomic strategies improved our ability to understand skin disorders, such as the structure and function of the microbiome in both diseased skin and its healthy states (83). In general, the proteomic technology contributed to the development of diagnosing skin disease and improved more sophisticatedly with high universality, practicality, and accuracy.

Initially, proteomics emerged as one of the technologies that gave a better explanation of the pathogenesis of disease within a simple verification. For example, the process of injured skin healing refers to a complex and highly regulated mechanism, such as growth factor (GF)-related clotting cascade, proteases-related necrotic tissue removal, and inflammation response-related repair and cell growth (84, 85). These findings have only described part of the pathogenesis of wound healing, whereas a broader perspective of proteins in the wound area is detected by proteomic technology. Proteomic analysis revealed the altered abundance of proteins in wound skin, suggesting the pathogenesis progress of myofibroblast contractility, extracellular matrix production, response to oxidative stress, and energy metabolism (86). Through mass spectrometric investigation, the progress of an antiangiogenic environment, excessive inflammation, and accelerated cell death have been found in exudates from chronic wounds (41). It has also found that neutrophil extracellular trap components were enriched in wound tissue, which may delay wound healing (87). Besides skin wounds, proteomic analysis has also been used in other skin diseases. As for chronic hand eczema (CHE), skin barrier dysfunction plays an essential role in the pathogenesis of CHE (88). An integrated quantitative proteomics approach was used to detect proteomic changes in the SARS-CoV-2-infected skin to provide a functional analysis of the response to microorganisms, apoptosis, and immune response, which leads to an insight into the virus-damaged skin response (89).

At present, in the research process of skin diseases, multi-omics combined analysis methods are more and more popular. Proteomics is often combined with genome, epigenome, transcriptome, metabolome, lipidome, exposure group, and microbiome for the exploration of various aspects of skin diseases. Take AD as an example. Genomics can help identify some of the underlying root causes of AD lesions, such as mutations in genes such as *BAT1*, *LCE3E*, *PCDH9*, and *PRR5l* (90–93). Proteomics can provide AD information in the form of phenomena during pathogenesis, namely, the protein profile of diseased tissue at specific time points (94, 95). Adipomics, microbiome, and exposome can also provide detailed information on various aspects (20). When these omics serve the same experiment or the same goal, they can reflect the pathogenesis of AD at multiple levels and broadly (96). Multi-omics are used more frequently and in depth in melanoma research. Such analysis can not only identify the multi-omics features that drive the molecular classification of melanoma but also provide precise guidance for subsequent treatment (97, 98). In fact, the advantage of multi-omics is that each project can help make up for the shortcomings of other projects, especially when one kind of omics is not enough to get a full picture of the disease process.

Recently, it has raised great attention on the mechanism of skin factors for skin diseases, such as UV damage, microorganism, and chemical agents. According to our data, proteomics plays an essential role in bridging the basic mechanism and risk factors. The research process generally uses risk factors to intervene on skin or cells. Second, proteomics has been used to analyze the proteome changes after the intervention. In addition, some studies further explored the effectiveness of specific agents against risk factors based on proteomic results. For instance, the research on diesel particulate extract conducted by Rajagopalan et al. investigated the treatment of vitamin E according to oxidant changes from proteomics (99). Most studies on microbial risk factors focus on screening virulence proteins from microorganisms *via* proteomics. For example, comparing the performance of RT4 and RT6 subtypes of *Cutibacterium acnes* in different living environments, triacylglycerol lipase and Christie–Atkins–Munch–Petersen (CAMP) factor were the critical virulence factors in pathogenesis (100). In addition, some studies examined infected skin or cells and have focused on pathogenic proteins derived from tissue cells (101). Using proteomics to study risk factors can provide detailed molecular changes and even mechanism predictions for the pathogenesis of particular diseases.

Interestingly, we have found the sample source differed from in various skin diseases. The most important source of samples for cancer was tumor cell lines cultured *in vitro* (59.302%). The advantage of using cell lines *in vitro* as research samples is that the results can reflect the characteristics of tumor cells in more detail and specially, which could exclude the interference of various other cells compared to skin tissue or tumor tissue sample. However, tissue samples, cell samples, and blood samples derived from patients or diseased mice are more realistic and comprehensive than *in vitro* cell lines in reflecting disease conditions and more conducive to studying the impact of tumor invasion, metastasis, and other deterioration processes. Moreover, cancer skin samples, primary fibroblasts (30), serum (31), and metastatic cancer tissue (32) have also been used in the study of progression and metastasis in cancer. Blood samples include serum, plasma, and biologically active substances and cells in the blood environment. These samples provide an accurate and detailed reflection of inflammatory changes in skin diseases. Based on these changes, biomarkers can obtain biomarkers (33), diseases can be distinguished (13, 34), and also pathogenesis can be studied with hub proteins (35, 36). In addition, the use of skin tissue samples and primary cell samples was also common. The advantage of skin tissue samples is that the proteomic results are closer to the actual pathological situation. In contrast, the primary cells can be used for detailed studies of single cells in the diseased site. In recent years, studies have begun to use blood samples and skin tissue samples from the same individual to obtain more comprehensive information on pathological changes (37, 38).

With the development of the enriched complex mechanism, proteomics helps gain a next-level insight or offer a breakthrough to guide further study. For example, CDK7 was identified by proteomics, and then its related function was proved by further experience to reveal that the downstream mechanism CDK7 could promote CD^4+^ T-cell activation and Th17/Th1 cell differentiation by regulating glycolysis, contributing to the pathogenesis of psoriasis (36). The phosphoproteomic analysis identified the signaling cascades downstream of FAT1 deletion. Then, combined with mechanistic studies, it jointly revealed that loss of function of FAT1 could activate a CAMK2-CD44-SRC axis to promote tumor initiation, progression, invasiveness, stemness, and metastasis (75). However, many more concerns remain for continued and improved research: (1) although the proteomic analysis could provide strong indications of expression level protein differences or the highlighted pathways, the hypothesized implications in several pathways or their co-network have to be confirmed not only in presentation but also their functions during the disease; (2) a specific or causative candidate has failed to identify for the certain skin disease, as well as the analysis mostly provided with the same inflammation factors or immune pathways in disease; (3) the different source of tissue (skin, guy, blood, urine, etc.) for proteomics may suggest systematic pathological changes during skin disorder, but it is still unclear to understanding their interface; (4) it is important to recognize the control populations included in the study, which should be appropriately matched in terms of other factors, and the subtype, the progression, and the interruption should be also provided in evidence; (5) strong evidence needs a large scale of samples limited to sample source and cost. However, there is no denying that proteomics leads to a well-founded hypothesis and a more systematic understanding of the pathogenesis of skin disorders.

Furthermore, a more accurate and systematic proteomic analysis has been contributed to clinical applications. According to our data, a majority of studies in proteomics utilized in skin disease focused on the specific proteins and their corresponding functions presented with a sensitive option for biomarker monitoring of diagnosis and disease progression evaluation. As for acute alopecia, Krt5 has been found as a novel biological marker for acute radiation symptoms *via* liquid chromatography/electrospray-ionization mass spectrometry (102) and a series of specific inflammation candidates have been reported for disease monitoring (38). Also other biomarkers were discovered through proteomics, such as acetaldehyde dehydrogenase 1 (ALDH1) for atopic dermatitis (103) and GPX5 for melanoma (104). Recently, the proteins in the circulatory system mirror an individual’s physiology, which could be utilized as a specific marker within a convenient sampling method (105). As plasma biomarkers’ specific analysis, a large-scale quantitative proteomic discovery helped identify the specific biomarker elafin for skin graft-versus-host disease and proved the specificity with enzyme-linked immunosorbent assay in samples from 492 patients (106). Interestingly, a series of specific proteins have been detected in the CD81-positive small extracellular vesicles through proteomics in melanoma, strengthening the circulating sEV as a systematic biomarker for early diagnosis for melanoma patients (107). Although multiple biomarkers have been termed, they tend to develop with high accuracy, specificity, and cost-effective combination of these candidates rather than a single indicator.

Also, proteomics has been utilized in the evaluation of the therapeutic strategy, and it is also benefited from a better understanding of the elusive mechanism of drug targets. As for the drugs for wound healing, it significant changes of abundance proteins have been found from the topical peri-wounding tissue after extracorporeal shock wave therapy (ESWT). After that, the related MAPK signaling might involve ESWT-enhanced diabetic wound healing (108). Also, through proteomic microarrays of the wound exudate, significantly higher levels of matrix metalloproteinases (MMPs) and lower levels of inflammation factors (e.g., CX3CL1, FLT-3 L, IL-1ra, IL-1a, IL-9, IL-2, IL-3) have been found to evaluate the therapeutic effect (109). The therapeutic effect also provided an optimal dose guiding drug therapy. For example, using collagen to treat aging could reduce the MMP-1, IL-1b, and IL-6 protein expression, especially in high concentration-treated in 160 mg/kg (110). Proteomics may lead to a systematic analysis, but Western blot and ELISA are still irreplaceable and classic methods, while part of certain proteins needs to be detected in skin disorders.

## Conclusion

Proteomics is a technique that provides the ability to identify changes in proteins at specific time points. It can provide key molecules, candidate biomarkers, and other information in the research of skin diseases. Based on this information, skin diseases can be more comprehensively explored through multichannel analysis methods, precise in-depth research, and the combination of multiple technologies. Various proteomic analyses have significantly contributed to a better understanding of the pathophysiology progress of skin, which is closely related to clinical applications. Although initial studies emerged to enrich skin proteomes and/or their related systemic disorder to establish comprehensive inventories, subsequent quantitative analysis paved the way to more in-depth studies addressing the detailed pathways underlying skin disease, guiding diagnosis, and therapy. Furthermore, spatial proteomics, multi-omics analysis, single-cell proteomics, and others may lead to a richer brainstorming for a deeper mechanism.

## Data Availability

The original contributions presented in the study are included in the article/Supplementary Material, further inquiries can be directed to the corresponding authors.
