# Supplementary material for: Proteomics as a tool to improve novel insights into skin diseases: what we know and where we should be going
Source: Front Surg. 2022 Oct 21;9:1025557. doi: 10.3389/fsurg.2022.1025557 (PMC9633964; doi:10.3389/fsurg.2022.1025557)
Supplement: Supplementary file 1 [file Table1.docx]

**Supplemental table 1.** Mechanism of skin cancer pathogenesis within proteomic analysis.

| **Type of disease** | **Sample** | **Highlighting mechanism** | **Depth mechanism** | **Ref.** |
| --- | --- | --- | --- | --- |
| Melanoma | Human melanoma tissue | Translation, proliferation, aggressiveness, etc. | OPN4 is an oncogene in melanoma. | de Assis et al., 2022 |
| Melanoma | Melanoma cell line | Cell death, atherosclerosis, PI3K, MAPK pathways | HMOX-1 was related to the resistant melanoma cells and its progress. | Zerfaoui et al., 2022 |
| Melanoma | Human [Primary (n=21), Metastatic (n=94)] | \ |  | Nowak et al., 2021 |
| Melanoma | Melanoma cell line | Metabolic, p53 pathway | \ | Yin et al., 2021 |
| Melanoma | Melanoma cell line | Metabolism/resistance, PI3K-AKT pathway, MAPK pathway. | \ | Giannopoulou et al., 2021 |
| Melanoma | Melanoma cell line | Ribosome biogenesis, gene. | FBOX32 was related to the melanoma progression and dissemination. | Habel et al., 2021 |
| Melanoma | Melanoma cell line | Migration, adhesion, skeleton. | GNAq/11 signaling completely abolished the ET-1 mediated rescue. | Schäfer et al., 2021 |
| Melanoma | Melanoma cell line | Adhesion, gene | \ | Chida et al., 2021 |
| Melanoma | Melanoma cell line | \ | \ | Francisquini et al., 2021 |
| Melanoma | Human [stages 0–IV melanoma (n=38), HCs (n=17)] | Metabolism, growth and/or maintenance, immune | APOA5, PON1, PON3 and CD14 was associated with better prognosis. | Paolino et al., 2021 |
| Melanoma | Human [patients (n=11)] | Translation, adhesion, gene, metabolic | \ | Gil et al., 2021 |
| Melanoma | Melanoma cell line | \ | AXL mediates the pro-invasive effects of ST3GAL1 in melanoma | Pietrobono et al., 2020 |
| Melanoma | Melanoma cell line | Tissue remodeling, immune regulation | \ | Chavanet et al., 2020 |
| Melanoma | Melanoma cell line |  | WDR74 is involved in cell cycle progression and apoptosis resistance. | Li et al., 2020 |
| Melanoma | Melanoma cell line | \ | \ | Neuditschko et al., 2020 |
| Melanoma | Melanoma cell line | Gene, cell morphology. | \ | Sanchez et al., 2020 |
| Melanoma | Human | \ | Bak scores were compared to Bak serum levels. | Longo et al., 2020 |
| Melanoma | Mice [WT (n=5), ADAM9-/- (n=5)] |  | Collagen type I density influences the proliferation. | Abety et al., 2020 |
| Melanoma | Human [P-M (n=24), P-NM (n=24)] | MAPK, translation, binding function | \ | Shapanis et al., 2020 |
| Melanoma | Human | \ | \ | Taylor et al., 2020 |
| Melanoma | Human [BRAF-MUT (n=110), BRAF-WT (n=122)] | \ | \ | Mukherjee et al., 2020 |
| Melanoma | Fibroblast | \ |  | Kaur et al., 2019 |
| Melanoma | Melanoma cell line | Adhesion, skeleton, PI3K/AKT, mTOR, MAPK pathway | Nestin affects cell proliferation and colony formation via PI3K/AKT and integrin signaling | Schmitt et al., 2019 |
| Melanoma | Melanoma cell line | \ | \ | Szász et al., 2019 |
| Melanoma | Melanoma cell line | Skeleton, ECM constituents, cell motility | \ | Liberato et al., 2018 |
| Melanoma | Melanoma cell line | \ | SerpinE2 is critical for melanoma invasion related to tumor progression. | Perego et al., 2018 |
| Melanoma | Melanoma cell line | Cell death, survival, growth, proliferation, cell Cycle, movement | \ | Rossi et al., 2018 |
| Melanoma | Melanoma cell line | Skeletal, ribosomal activity, ROS, angiogenesis | \ | Wilking-Busch et al., 2018 |
| Melanoma | Melanoma cell line | Energetic metabolism, structure and folding | NMP1, and PARK7 were correlated with migration ability. | Cecconi et al., 2018 |
| Melanoma | Melanoma cell line | \ | \ | Ngeow et al., 2018 |
| Melanoma | Melanoma cell lines | Gene, adhesion, metabolism | \ | Hoja-Łukowicz et al., 2018 |
| Melanoma | Melanoma cell line | Metabolizes, gene, cell cycle, PI3K-Akt pathway | \ | Konstantakou et al., 2017 |
| Melanoma | Human [Melanoma (n=10)] | Gene, adherent, metabolic. | \ | Welinder et al., 2017 |
| Melanoma | Melanoma cell line | Skeleton, energy production, etc. | Increased FAK1 signaling mediated increased invasiveness in BRAFi resistant cells | Sharma et al.,2016 |
| Melanoma | Melanoma cell line | Skeleton, RTK signaling, etc. | \ | Fedorenko et al., 2016 |
| Melanoma | Melanoma cell line | PI3K/AKT/rS6 pathway, transcription. | \ | Posch et al., 2016 |
| Melanoma | Human [Primary (n=5), Metastatic (n=9)] | \ | \ | Dowling et al., 2016 |
| Melanoma | Melanoma cell line | Adhesion, skeleton, FAK, integrin signaling | Ligand-independent EphA2 signaling is AKT-dependent, and drives the adoption of an invasive phenotype | Paraiso et al., 2015 |
| Melanoma | Human [Primary (n=18), Metastatic (n=12)] | \ | MDA-9 and GRP78 related to metastatic melanoma progress. | Guan et al., 2015 |
| Melanoma | Human | GTPase regulator activity, glycolysis | \ | Welinder et al., 2015 |
| Melanoma | Melanoma cell line | Formyl peptide receptor | \ | Boudhraa et al., 2014 |
| Melanoma | Human [Melanoma (n=311)] | Transcription, DNA repair | \ | Uzdensky et al., 2014 |
| Melanoma | Melanoma cell line | Metabolic activity, protein binding | Validation of nestin and vimentin can help predict melanoma aggressiveness | Qendro et al., 2014 |
| Melanoma | Melanoma cell line | MAPK, PI3K/AKT pathways | \ | Fedorenko et al., 2014 |
| Melanoma | TIBCs | Metabolism, cell death | \ | Maurer et al., 2014 |
| Melanoma | Human | \ | \ | Schowalter et al., 2014 |
| Melanoma | Human [Invasive melanoma (n=20), metastatic lymph node (n=5)] | POSTN-integrin/P44/42MAPK in proliferation | POSTN-integrin/P44/42MAPK in proliferation proved by WB, qPCR. | Kotobuki et al., 2014 |
| Melanoma | Human [PP (n=14), GP (n=19)] | Metabolism, angiogenesis, apoptosis, immune response. | \ | Mactier et al., 2014 |
| Melanoma | Human [Benign nevi (n=25), Primary (n=12), Metastatic (n=24)] | MAPK signaling, adhesion, skeleton | \ | Byrum et al., 2013 |
| Melanoma | Human | MAPK, integrin signaling etc. | \ | Murillo et al., 2012 |
| Melanoma | Human (n=18) | Morphology, detoxification | \ | Gao et al., 2012 |
| Melanoma | Melanoma cell line | \ | Panx1 was related to melanoma reversion. | Penuela et al., 2012 |
| Melanoma | Melanocytes and melanoma cell line | \ | \ | Caputo et al., 2011 |
| Melanoma | Melanoma cell line | Cell death, growth, proliferation, metastasis, adhesion | \ | Rocco et al., 2011 |
| Melanoma | Melanoma cell line | Cell death, growth, tumorigenesis | \ | Han et al., 2010 |
| Melanoma | Melanocytes and melanoma cell line | \ | \ | Paulitschke et al., 2009 |
| Melanoma | Human [Melanoma (n=31), HCs (n=10)] | \ | Plasma PPBP levels were correlated with the survival time | Takikawa et al., 2009 |
| Melanoma | Melanoma cell line | \ | \ | Baruthio et al., 2008 |
| Melanoma | Melanoma cell line | \ | \ | Seabra et al., 2007 |
| Melanoma | Melanoma cell line | \ | \ | Carta et al., 2005 |
| Melanoma | Melanoma cell line | \ | \ | Bernard et al., 2003 |
| NMSCs | Epidermoid carcinoma cells lines | Differentiation, adhesion, junction, etc. | \ | Paulitschke et al., 2015 |
| Skin cancer | Human [patients (n=17)] | Cell death, cell architecture, adhesion, motility | \ | Tramutola et al., 2020 |
| Skin cancer | Epidermal cell lines | Adhesion, skeleton organization, etc. | \ | Lee et al., 2017 |
| Skin cancer | Mice | \ | S100A8 and S100A9 protein levels were proved as skin tumor promoters. | Shen et al., 2012 |
| Skin cancer | UVB irradiated NHEK | Adhesion, skeleton maintenance, misfolded proteins removal, cell growth, tumor suppression | \ | Perluigi et al., 2010 |
| CTCL | Human [CTCL (n=10), psoriasis (n=24)] | \ | \ | Méhul et al., 2019 |
| Ear cholesteatoma | Human cholesteatoma tissue | \ | CRT and AnxA2 downregulation are seen in cholesteatoma compared to retroauricular skin. | Chang et al., 2022 |
| CC | Human [CC (n=6), AC (n=2), RAS (n=6)] | \ | FKH 5-3 and titin localized in membrane and cytoplasm in layers of congenital cholesteatoma keratinocytes. | Shin et al., 2015 |
| CMM | Human [Chemotherapy responders (n=5), non-responders (n=5)] | Skeleton, interaction | \ | Azimi et al., 2014 |
| Cutaneous Iymphoma | Human sera | \ | \ | Forgber et al., 2009 |
| MCC | MCC cell lines | Metabolic, protein folding, transductions. | \ | Kotowski et al., 2019 |
| MCC | MCC cell lines | \ | \ | Konstantinell et al., 2016 |

(NMSC: Nonmelanoma skin cancers; SCC: Squamous cell carcinoma; CMM: Cutaneous malignant melanoma; CTCL: Cutaneous T-cell lymphomas; MCC: Merkel cell carcinoma; HCs: Human controls; TIBCs: Tumor-infiltrating B cells; WT: Wide type; P-M: Primary melanomas which had metastasised; P-NM: Primary melanomas that had not metastasised; MUT: Mutation; PP: Poor prognosis; GP: Good prognosis; AK: Actinic keratosis; UVB: Utraviolet B; NHEK: Normal human epithelial keratinocyte; AC: Acquired cholesteatoma; CC: Congenital cholesteatoma; RAS: Retroauricular skin; MAPK: Mitogen-activated protein kinase; PI3K: Phosphatidylinositol-4,5-bisphosphate 3-kinase; FAK: Focal adhesion kinase; RTK: Receptor tyrosine kinase; ECM: Extracellular matrix; ROS: Reactive oxygen species; mTOR: mechanistic target of rapamycin; JNK/SAPK: c-Jun N-terminal kinase/stress-activated protein kinase; CDC42: Cell division cycle 42; RAP1: Role of ras-associated protein 1; EGFR: Epidermal growth factor receptor; Panx1: Pannexin 1; AKT: Protein kinase B; BRAFi: B‐Raf proto‐oncogene, serine/threonine kinase inhibitor; NMP1: Nucleophosmin 1; PARK7: Parkinson's disease protein 7; ST3GAL1: ST3 beta-galactoside alpha-2,3-sialyltransferase 1; FBOX32: F-box protein 32; ET-1: Endothelin-1; APOA5: Apolipoprotein 5; PON: Paraoxonase; HMOX-1: Heme oxygenase-1; POSTN: Periostin; K17: Keratin 17; NF-κB: Nuclear factor-kappaB; NNMT: N-nicotinamide methyltransferase; YAP1: Yes-associated protein 1; SOX2: SRY-box transcription factor 2; HMGCS1: 3-hydroxy-3-methylglutaryl-CoA synthase 1; FKH 5-3: Forkhead transcription activator homolog; CRT: Calreticulin; AnxA2: Annexin A2)
