# Supplementary material for: Proteomics as a tool to improve novel insights into skin diseases: what we know and where we should be going
Source: Front Surg. 2022 Oct 21;9:1025557. doi: 10.3389/fsurg.2022.1025557 (PMC9633964; doi:10.3389/fsurg.2022.1025557)
Supplement: Supplementary file 2 [file Table2.docx]

**Supplemental table 2.** Mechanism of inflammatory skin disease pathogenesis within proteomic analysis.

| Type of disease | Sample | Highlighting mechanism | Depth mechanism | Ref. |
| --- | --- | --- | --- | --- |
| AD | Human | \ | \ | Koch et al., 2022 |
| AD | Human [AD (n=12), HCs (n=13)] | Localization, regulation of biological quality, platelet activation, etc. | \ | Chang et al., 2021 |
| AD | Human [AA (n = 35), HCs (n = 36), Psoriasis (n = 19), AD (n = 49)] | Atherosclerosis signaling, immune pathways, cardiovascular pathway | \ | Glickman et al., 2021 |
| AD | Human [AD (n=4), HCs (n=7), Spontaneously healed AD (n=4)] | \ | \ | Rindler et al., 2021 |
| AD | Human [AD (n=34), HCs (n=20)] | \ | Insufficiency of IL-37 leads to dysregulation of serum protein and skin disruption in AD. | Hou et al., 2021 |
| AD | Human [AD (n=8), HCs (n=8)] | Biological regulation, cellular component organization, etc. | \ | Morelli et al., 2021 |
| AD | Human [AD (n=20), HCs (n=28)] | \ | \ | Pavel et al., 2020 |
| AD | Skin suction blisters and skin | \ | \ | Rojahn et al., 2020 |
| AD | Human [18-40 years old (n=26), 41-60 years (n=24), >60 years (n=21), HCs (n=37)] | Th1/Th2 differentiation, IL-4-mediated signaling, IL-5-mediated signaling, etc. | \ | He et al., 2020 |
| AD | Human [AD + FA (n=21), AD (n=19), HCs (n=22)] | Inflammatory response, glycolysis, oxidative stress response | \ | Goleva et al., 2020 |
| AD | Human [AD (n=20), HCs (n=7)] | \ | \ | Umayahara et al., 2020 |
| ACD | Mice TGs | Response to stimulus, metabolic process, immune system process, etc. | \ | Su et al., 2020 |
| AD | Human [AD (n=76), HCs (n=39)] | \ | \ | Leonard et al., 2020 |
| AD | Human | \ | \ | Yin et al., 2019 |
| AD | Human [AD pediatric (n=30), Healthy pediatric (n=19), AD adult (n=58), Healthy adult (n=18)] | \ | \ | Brunner et al., 2019 |
| AD | Human [HCs (n=84),severe AD (n=50), moderate AD (n=123)] | \ | \ | Mikus et al., 2019 |
| AD | Human [HC (n=10), AD (n=20), CD (n=10), AD and CD (n=10), Psoriasis (n=12)] | \ | \ | Wang et al., 2017 |
| AD | Human [Psoriasis (n=22), AD (n=59), HCs (n=18)] | \ | \ | Brunner et al., 2017 |
| ACD | Dendritic-like cell line | Cell signaling, transcriptional regulation, protein transport, ubiquitination, etc. | MLK is one of FITC targets leading the specific protein haptenation and the subsequent pathway of dermal dendritic cells activation. | Guedes et al., 2017 |
| AD | Spleen and thymus Treg cells | \ | \ | Lee et al., 2016 |
| AD | AD-NC/Nga mice | \ | \ | Kawasaki et al., 2014 |
| AD | Human [EH− (n=18), EH+ (n=17), non-atopic controls (n=6)] | Skin barrier, generation of natural moisturizing factor | \ | Broccardo et al., 2011 |
| AD | Human [AD (n=8), HCs] | \ | \ | Kim et al., 2008 |
| AD | Human [ADe (n=14), ADi (n=10), HCs (n=14)] | \ | \ | Park et al., 2007 |
| AD | Human [ADe (n=14), ADi (n=10), HCs (n=14)] | \ | \ | Park et al., 2007 |
| AD | Human [ADe, ADi, HCs] | \ | \ | Park et al., 2006 |
| AD | Human [ADe, ADi, HCs] | \ | \ | Park et al., 2004 |
| Psoriasis | Human [AA (n = 35), HCs (n = 36), Psoriasis (n = 19), AD (n = 49)] | Atherosclerosis signaling, immune pathways, cardiovascular pathway | \ | Glickman et al., 2021 |
| Psoriasis | Human [Psoriasis (n=3), HCs (n=3)] | \ | CDK7 can modulate CD4+ T cell activation and Th17/Th1 cell differentiation contributing to the pathogenesis of psoriasis. | Lin et al., 2021 |
| Psoriasis | Human [Psoriasis (male,n=2; female,n=3), HCs (male,n=3; female,n=2)] | Response to estrogen | \ | Sobolev et al., 2021 |
| Psoriasis | Human | Cell adhesion and junction, immune system process, extracellular matrix, immune dysfunction, etc. | \ | Li et al., 2021 |
| Psoriasis | Human [Psoriasis (n=45)] | Immune response, defence response, regulation of endopeptidase activity, etc. | \ | Wang et al., 2021 |
| Psoriasis | Human [PsA (n = 20), Psoriasis (n = 18), Arthritis (n = 19), HCs (n = 20)] | \ | \ | Leijten et al., 2021 |
| Psoriasis | Human [Psoriasis (n=12), HCs (n=12)] | \ | \ | Qiu et al., 2020 |
| Psoriasis | Human [Psoriasis (n=16), HCs (n=15)] | Signal transduction, transport, proteolysis, small GTPase mediated signal transduction | \ | Zhou et al., 2020 |
| Psoriasis | Human [Psoriasis (n=11), HCs (n=11)] | Immune response, cell proliferation, metabolism-related pathways | \ | Li et al., 2020 |
| Psoriasis | Human [Psoriasis (n=5), HCs (n=5)] | Transcription/translation processes, protein folding, glycolysis/ATP synthesis, antioxidant response, inflammation | \ | Gęgotek et al., 2020 |
| Psoriasis | Human [Psoriasis (n=45)] | Cell development, Cell cycle, Pathogenic Escherichia coli infection, PI3K-AKT signaling pathway, etc. | \ | Ge et al., 2019 |
| Psoriasis | Human | Regulation of immune effector process, acute inflammatory response, Wnt signaling pathway, NF-kB signaling pathway | Exosomes from IMQ-treated epidermis exacerbate skin inflammation in IMQ-induced psoriasis-like mice | Jiang et al., 2019 |
| Psoriasis | Human [Psoriasis (n=6), HCs (n=6)] | \ | \ | Gęgotek et al., 2019 |
| Psoriasis | Human [CTCL (n=10), Psoriasis (n=24)] | \ | \ | Méhul et al., 2019 |
| GPP | Human [HCs (n = 10), GPP (n = 10)] | Cell growth and maintenance, signaling transduction, metabolism, cell communication, immune response | Recombinant OLFM4 induced high expression and secretion of chemokines in keratinocytes | Shao et al., 2019 |
| Psoriasis | Human [Psoriasis (n=6), HCs (n=6)] | \ | \ | Gęgotek et al., 2018 |
| Psoriasis | Human [Psoriasis (n = 31), HCs (n = 32)] | Cell respiration, nucleobase-containing compround, DNA metabolic process, oxidative phosphorylation, tricarboxylic acid cycle, NF-κB pathway, RAS signaling pathway | \ | Li et al., 2018 |
| Psoriasis | Human [Psoriasis (n=20), HCs (n=10)] | \ | \ | Garshick et al., 2017 |
| Psoriasis | Human [HC (n=10), AD (n=20), CD (n=10), AD + CD (n=10), Psoriasis (n=12)] | \ | \ | Wang et al., 2017 |
| Psoriasis | Human | Epidermis development, keratinocyte differentiation, glycolysis, regulation of apoptosis, etc. | \ | Méhul et al., 2017 |
| Psoriasis | Mice [KC-Tie2 (n=3), controls (n=3)] | \ | \ | Lundberg et al., 2015 |
| Psoriasis | Human [Psoriasis (n=14)] | Defense response to virus, response to type I interferon, cell death, etc. | \ | Swindell et al., 2015 |
| Psoriasis | White human [Psoriasis vulgaris (n=20), HCs (n=16)] | \ | \ | Fattahi et al., 2014 |
| Psoriasis | Human[Psoriasis (n=19)] | \ | Genetic deletion of S100A9 reduced expression of complement factor C3 and its activation partner CFB, as well as IL-1b, thereby preventing the psoriasis-like disease. | Schonthaler et al., 2013 |
| Psoriasis | Human[Psoriasis (n=4)] | \ | \ | Williamson et al., 2013 |
| Psoriasis | Human[Psoriasis (n=15), HCs (n=15)] | \ | Altered HDL composition is linked to impaired cholesterol efflux capacity. | Holzer et al., 2012 |
| Psoriasis | Human primary cells | \ | \ | Ariza et al., 2011 |
| Psoriasis | Huamn [Psoriasis (n=5), HCs (n=40)] | Regulation of apoptosis, inflammatory respons, enzyme activity, protein binding activity, etc. | Immunoblotting using GSTP1, SFN and PRDX2 antibodies. | Ryu et al., 2011 |
| Psoriasis | Human [Psoriasis (n=3), HCs (n=3)] | Cell adhesion, chemotaxis, apoptosis, immune response p38 MAPK, NF-kb signaling pathway | \ | Piruzian et al., 2010 |

(Abbreviation: AD: Atopic dermatitis; ACD: Atopic contact dermatitis; HCs: Human controls; AA: Alopecia areata; TGs: Trigeminal ganglions; GPP: Generalized pustular psoriasis; CD: Contact dermatitis; EH: Eczema herpeticum; ADe: Extrinsic AD; ADi: Intrinsic AD; PsA: Psoriatic arthritis; CTCL: Cutaneous T-cell lymphomas; PI3K: Phosphatidylinositol-4,5-bisphosphate 3-kinase; AKT: Protein kinase B; NF-kB: Nuclear transcription factor-kappa B; MAPK: Mitogen-activated protein kinase; FITC: Fluorescein isothiocyanate; MLK: Mixed-lineage protein kinase; CDK: Cyclin‐dependent kinase; IMQ: Imiquimod; OLMF4: Olfactomedin 4; CFB: Complement factor B; HDL: High density lipoprotein; GSTP1: Glutathione S transferase 1; PRDX2: Peroxiredoxin 2)
