# Supplementary material for: Proteomics as a tool to improve novel insights into skin diseases: what we know and where we should be going
Source: Front Surg. 2022 Oct 21;9:1025557. doi: 10.3389/fsurg.2022.1025557 (PMC9633964; doi:10.3389/fsurg.2022.1025557)
Supplement: Supplementary file 3 [file Table3.docx]

**Supplemental table 3.** Risk factors of skin disease analyzed with proteomics.

| **Risk factor** | **Type of diorder** | **Highlighting mechanism** | **Options for risk factors** | **Ref.** |
| --- | --- | --- | --- | --- |
| UVA | Skin damages | Intracellular transport, cellular localization, etc. | \ | Valerio et al., 2022 |
| UVB | Skin aging | Cell apoptosis, cell cycle, etc. | Human umbilical cord mesenchymal stem cell-conditioned medium | Zou et al., 2022 |
| UVA, UVB | Skin damages | \ | \ | Zhao et al., 2022 |
| UVA, UVB | Skin damages |  | CBD | Gan et al., 2021 |
| UV | AK/SCC | Chaperoning and stress response, protein folding/degradation, apoptosis/survival | \ | Tramutola et al., 2020 |
| UVA | Skin damages | eIF2 stress signaling, PKA pathway protein ubiquitination, DNA damage response signaling | \ | Narzt et al., 2019 |
| UVA, UVB | Skin damages | \ | \ | Wang et al., 2019 |
| UVA, UVB | Skin damages | \ | Rutin + Ascorbic acid | Gęgotek et al., 2019 |
| UVA, UVB | Skin damages | Inflammation, the antioxidant response, apoptosis | Rutin | Gęgotek et al., 2018 |
| UVB | Skin damages | \ | Baicalin | Li et al., 2017 |
| UVB | Skin damages | Protein binding, hydrolase activity, etc. | \ | Liu et al., 2012 |
| UVB | Skin damages | Protein folding, signaling, cytoskeleton regulation and protein biosynthesis | \ | Wu et al., 2012 |
| UVA | Skin photoaging and photocarcinogenesis | \ | Antioxidant N-acetyl-L-cysteine | Lamore et al., 2010 |
| UVB | Acute photodamage, photoaging and skin cancer | \ | \ | Yan et al., 2010 |
| UV | DNA lesions | \ | \ | Yuasa et al., 2006 |
| UV | Skin damages | \ | \ | Hensbergen et al., 2005 |
| Arsenic | \ | \ | \ | Berglund et al., 2020 |
| Arsenic | \ | Redox metabolism, beta-alanine metabolism, TCA cycle, etc. | \ | Zhou et al., 2017 |
| Arsenic | Arsenic keratosis | Structural molecule, catalytic activity, binding activity, etc. | \ | Guo et al., 2016 |
| Arsenic | \ | \ | \ | Udensi et al., 2014 |
| Irradiation | Skin damages | Signal transduction, regulation of biological process, cell organization and biogenesis | \ | Yang et al., 2020 |
| X-ray irradiation | \ | Proteasome, neurotrophin signaling, leukocyte transendothelial migration, focal adhesion pathway | Bortezomib | Wang et al., 2016 |
| Low-Dose Ionizing Radiation | Skin damages | Protein Ubiquitination Pathway, EIF2 Signaling, etc. | \ | Hengel et al., 2014 |
| Ionizing radiation | \ | Protein binding, cell signalling, cell movement, epidermis development, endoplasmic reticulum stress response, inflammation, free radical scavenging | \ | Zhang et al., 2014 |
| Localized irradiation | Irradiation injury | Negative regulation of endopeptidase activity, etc. | \ | Chaze et al., 2013 |
| Ionizing radiation | Skin damages | PKA signaling pathway, MAP kinase signaling pathway, etc. | \ | Yang et al., 2010 |
| Ionizing radiation | Skin damages | \ | \ | Guipaud et al., 2007 |
| Er:YAG laser | \ | Cytoskeleton, chaperone, growth and differentiation, protein enzymes | \ | Pan et al., 2010 |
| Heavy metals | \ | \ | \ | Zhang et al., 2010 |
| Hexavalent Chromium | Cr(VI)-induced damages | Cholesterol biosynthesis, inflammatory response, GPCR signaling pathways, suppression of selenoproteins | \ | Guo et al., 2013 |
| Hexavalent Chromium | Cr(VI)-induced damages | Cell redox status, immunology, cell proliferation | \ | Pan et al., 2009 |
| Lead compounds | \ | \ | \ | Pan et al., 2010 |
| Sulfur mustard | \ | Organelle inner membrane, endocytic vesicle, etc. | \ | Jamshidi et al., 2022 |
| Sulfur mustard | \ | \ | \ | Steinritz et al., 2013 |
| Sulfur mustard | \ | \ | \ | Mol et al., 2008 |
| Cadmium | NO biosynthesis in human skin fibroblast cells | Glutathione metabolism, glycolysis and gluconeogenesis, pyruvate metabolism, etc | \ | Prins et al., 2014 |
| Jellyfish venom | \ | Immune response, cell survival and development, biological adhesion and localization, etc. | \ | Choudhary et al., 2021 |
| PA1 | Respiratory allergies | \ | \ | Khong et al., 2021 |
| Snakebite envenomation | Skin blistering | Platelet degranulation, negative regulation of endopeptidase activity, innate immune response, etc. | \ | Macêdo et al., 2019 |
| TiO2-NPs | ROS generation,increased inflammatory response | Cell cycle regulation, gene expression, metabolic processes, etc. | \ | Montalvo-Quiros et al., 2019 |
| Collagen VII deficience | DEB | \ | \ | Thriene et al., 2018 |
| Cosmetic peroxide | Oxidative damage | \ | \ | Grosvenor et al., 2018 |
| DPE, DPE vapor | Oxidative stress response | \ | Vitamin E | Rajagopalan et al., 2018 |
| Sodium arsenite | \ | NF-κB signaling, ERK signaling, NRF2-KEAP1 pathway, | \ | Mir et al., 2017 |
| SSL | Skin cancer | MAPK, AKT-mTOR and IGFR pathways, cell death/apoptosis-related pathways | \ | Einspahr et al., 2017 |
| Cigarette smoke | \ | Oxidative stress, skin integrity maintenance, anti-inflammatory responses | Vitamin E | Rajagopalan et al., 2016 |
| dNCOs | \ | \ | \ | Nayak et al., 2014 |
| TCDD | Cancer | \ | \ | Hu et al., 2013 |

(Abbreviation: UV: Utraviolet; PA1: Phthalic anhydride 1; TiO2-NPs: Titanium dioxide nanoparticles; DPE: Diesel particulate extract; SSL: Solar stimulated light; TCDD: 2,3,7,8-tetrachlorodibeno-p-dioxin; Er:YAG: Erbium:yttrium-aluminum-garnet; SCC: Squamous cell carcinoma; AK: Actinic keratosis; ROS: Reactive oxygen species; DEB: Dystrophic epidermolysis bullosa; PKA: Protein kinase; NF-κB: Nuclear factor-kappaB; NFR2: Nuclear factor erythroid 2 -related factor 2; KEAP1: Kelch-like erythroid cell-derived protein with CNC homology-associated protein 1; MAPK: Mitogen-activated protein kinase; AKT: Protein kinase B; mTOR: mechanistic target of rapamycin; IGFR: Insulin-like growth factor receptor; TCA: Trichloroacetic acid; EIF2: Eukaryotic initiation factor 2; GPCR: G protein-coupled receptor; MAP: mitogen-activated protein; CBD: Cannabidiol.)
