# Supplementary material for: Proteomics as a tool to improve novel insights into skin diseases: what we know and where we should be going
Source: Front Surg. 2022 Oct 21;9:1025557. doi: 10.3389/fsurg.2022.1025557 (PMC9633964; doi:10.3389/fsurg.2022.1025557)
Supplement: Supplementary file 4 [file Table4.docx]

**Supplemental table 4.** Option for treating skin disease analyzed with proteomics.

| **Type of disease** | **Option** | **Sample** | **Enriched mechanism** | **Depth mechanism** | **Ref.** |
| --- | --- | --- | --- | --- | --- |
| Melanoma | IACS + STN | Melanoma cell lines | MAPK pathway, AKT pathway | \ | de Groot et al., 2022 |
| Melanoma | CHI3L1 | Melanoma cell lines | \ | CHI3L1 is able to promote angiogenesis through the release of extracellular matrix-bound pro-angiogenic factors. | Ramos-Espinosa et al., 2021 |
| Melanoma | Meso-Xanthin | Melanoma cell lines | Apoptosis, regulation of transcription, immune response, MAPK pathway, etc. | \ | Saburina et al., 2021 |
| Melanoma | Anti-CTLA-4, Anti-PD-1, MEKis, BRAFis | Human plasma [ICIs treatment (n=24), MAPKi treatment(n=24)] | Immune response, acute inflammation | \ | Babačić et al., 2020 |
| Melanoma | Afatinib + Crizotinib | Melanoma cell lines | RNA splicing, ribonucleoprotein complex assembly, mTOR signaling, etc. | \ | Das et al., 2020 |
| Melanoma | MMTC | Melanoma cell lines | \ | \ | Choi et al., 2020 |
| Melanoma | NRF2 silence | Melanoma cell lines | EMT, coagulation, ROS pathway | \ | Weitzenböck et al., 2020 |
| Melanoma | Dabrafenib, Trametinib, SCH772984 | Melanoma cell lines | \ | \ | Santini et al., 2019 |
| Melanoma | Theophylline | Melanoma cell lines | mRNA processing, cell proliferation, etc. | \ | Cordella et al., 2019 |
| Melanoma | TIL-based or Anti-PD1 immunotherapy | Human tumor tissue [TIL (n=42), Anti-PD1 (n=74)] | TCA cycle, fatty acid oxidation, MAPK signaling pathway, etc. | \ | Harel et al., 2019 |
| Melanoma | MAPKi | Melanoma cell lines | Cell adhesion, ECM receptor interaction, etc. | \ | Paulitschke et al., 2019 |
| BRAF V600E mutant melanoma | Vemurafenib + XL888 | Human tumor biopsies [30 mg XL888, 45 mg XL888, 90 mg XL888, 135 mg XL888] | \ | \ | Eroglu et al., 2018 |
| Melanoma | Dabrafenib | Melanoma cell lines | \ | **1.**Suppression of NEK9 inhibits the growth of *RAS*‐mutant cells and modulates the expression of p21 and pCHK1; 2.NEK9 silencing leads to senescence associated with decreased CDK4 expression; 3.Dabrafenib‐mediated CDK16 inhibition enhances the cell cycle effects of NEK9 inhibition. | Phadke et al., 2018 |
| Melanoma | BSE-SFN | Human nevus tissues | \ | \ | Tahata et al., 2018 |
| Melanoma | NSC697923(small-molecule inhibitor of UBE2N) | Melanoma cell lines | \ | FRA1 gene silencing decreased pMEK, pERK, and SOX10 expressions | Dikshit et al., 2018 |
| Melanoma | Trametinib | Melanoma cell lines | MAPK pathway | Combining MEK inhibition with selective targeting of c-JUN and/or FAK, sensitised resistant cell lines and significantly enhanced treatment efficacy | Rožanc et al., 2018 |
| Melanoma | BETis + MEKis | Melanoma cell lines | \ | \ | Echevarría-Vargas et al., 2018 |
| Melanoma | Cisplatin, Dacarbazine, Temozolomide, Tunicamycin | Melanoma cell lines | \ | PHB is necessary for cell adaptation against cellular stress. | Tortelli et al., 2017 |
| Melanoma | PA | Melanoma cell lines | Regulation of apoptosis, cell cycle, etc. | \ | Lai et al., 2015 |
| Muco-cutaneous tumors | PDT, AlS2Pc | Dysplastic oral keratinocyte cell line | \ | \ | Matei et al., 2014 |
| CTCL | SAHA | SH-SY5Y cells | Cellular respiration, acetyl-CoA metabolism, TCA cycle, valine degradation, etc. | \ | Xu et al., 2014 |
| Melanoma | Volasertib | Melanoma cell lines | Catalytic activity, binding, metabolic process | \ | Cholewa et al., 2014 |
| Melanoma | Tenovin-1 | Melanoma cell lines | Cellular metabolism, cell death, cell cycle, apoptosis signaling, etc. | Tenovin-1 inhibition of SIRT1 resulted in a downregulation of BUB3, BUB1 and BUBR1. | Singh et al., 2014 |
| Skin cancer | Mancozeb | Swiss albino mice skin | \ | S100A6 and S100A9 regulated ERK1/2 signaling underlies the mancozeb-induced neoplastic potential. | Tyagi et al., 2011 |
| Melanoma | M8 | Melanoma cell lines | \ | \ | Paulitschke et al., 2010 |
| Skin cancer | Glyphosate | Mice skin [Glyphosate,TPA, DMBA, Controls] | Apoptosis and growthinhibition, anti-oxidation, etc. | \ | George et al., 2010 |
| Melanoma | Surgery | Human serum | \ | \ | Greco et al., 2009 |
| Skin cancer | TPA | Mouse skin | \ | \ | Shen et al., 2007 |
| Wound | Angio^PRP^ | Human skin | Inflammatory response, wound healing, etc. | \ | Erratico et al., 2022 |
| Wound | Mesenchymal stromal cells derivatives | Human skin | Extracellular matrix organization, biological adhesion, etc. | \ | Arango-Rodríguez et al., 2022 |
| Wound | ANBP | Mice skin | Cellular process, Single-organism process, etc. | \ | Zhou et al., 2022 |
| Chronic venous leg ulcers | HSAM | Human wound exudate [Ulcers (n=15)] | \ | \ | McQuilling et al., 2021 |
| Wound | OCT | Human skin [TS (n = 5), HCs (n = 3)] | IL-6 ,MMP1/2/3/9 signalng, etc. | \ | Seiser et al., 2021 |
| Wound | Topical ALA-induced PDT | C57BL/6(C57) mice EpSCs | \ | \ | Yang et al., 2021 |
| Wound | ESWT | Wistar rodents skin | \ | \ | Chen et al., 2020 |
| Wound | CBD, H2O2 | Human epidermal keratinocytes | Proteasomal activity, protein folding processes, protein biosynthesis, etc. | \ | Atalay et al., 2020 |
| Burn wound | ADSCs | SD rats skin | \ | \ | Zhou et al.,2019 |
| Wound | HA | Human skin | \ | \ | Nyman et al., 2019 |
| Wound | NO | Human foreskin tissue | \ | \ | Zhan et al., 2018 |
| Wound | Extracorporeal shock wave | Rats serum | \ | \ | Yang et al., 2014 |
| Wound | GGA | Human epidermal keratinocytes | \ | \ | Isoir et al., 2013 |
| Wound | Platelet lysate | Human skin | Cell proliferation, cytoskeleton organization, protein metabolism | \ | Cipriani et al., 2009 |
| Psoriasis | JYG | Mice skin [Controls (n=4); Psoriasis (n=4), Psoriasis + JYG (n=4), Psoriasis + MTX (n=4)] | Estrogen signaling pathway, cholesterol metabolism, fat digestion and absorption, PPAR signaling pathway, etc. | \ | Song et al., 2022 |
| Psoriasis | YWS | Mice skin [Controls (n=8); Psoriasis (n=8), Psoriasis + YWSH (n=8), Psoriasis + YWSL (n=8)] | Butyrate metabolism pathway, leukocyte transendothelial migration, etc. | \ | Su et al., 2022 |
| Psoriasis | LLDT-8 | Mice skin [Untreated (n=8), Vehicle (n=8), LLDT-8 (n=8)] | Keratinization; IL-36 pathway; Immune system process; Neutrophil  migration; Inflammatory response | \ | Qi et al., 2021 |
| Psoriasis | Sericin extracted from *Bombyx mori* cocoon | Mice skin [Vaseline (n=5); 2.5% sericin cream (n=5), 5% sericin cream (n=5), 10% sericin cream (n=5); 0.1% betamethasone (n=5); 3μg/ml calcitriol ointment (n=5)] | Th17 cell differentiation, JAK-STAT signaling pathway | \ | Rujimongkon et al., 2021 |
| Psoriasis | Taxifolin | Mice skin | Regulation of protein activation cascade, MHC class II protein complex, etc. | \ | Di et al., 2021 |
| Psoriasis | Secukinumab | Human serum [Psoriasis (n=8), HCs (n=8)] | \ | BD-2 levels correlated well with IL-17A levels and Psoriasis Area and Severity Index scores | Kolbinger et al., 2017 |
| Psoriasis | Efalizumab | Human skin [Responder (n=5), Non-responder (n=1)] | \ | \ | Bonnekoh et al., 2007 |
| Psoriasis | Efalizumab | Human skin [Psoriasis, HCs] | \ | \ | Bonnekoh et al., 2007 |
| Photo-ageing | Low-level red plus near infrared lights | Human fibroblasts and skin | \ | \ | Li et al., 2021 |
| Aging | Poly- and Oligosaccharide Ulva sp. Fractions | Human dermal fibroblasts | \ |  | Fournière et al., 2021 |
| Photoaging | SMHF, SMEAF | Human keratinocyte cell line | Redox system, DNA repair and maintenance, RNA transcription to translation, etc. | \ | Mahendra et al., 2021 |
| Photoaging | Carnosine | Mice skin [Controls (n=4), UVA (n=4), Vehicle + UVA (n=4), Carnosine + UVA (n=4)] | Calcium signaling, mitochondrial function, sirtuin expression | \ | Radrezza et al., 2021 |
| Photoaging | n-3 PUFA | Mice skin [NC (n=5), NF (n=5), UC (n=5), UF (n=5)] | UF vs. UC: Lipid metabolism, etc.; UC vs. NC: Establishment of the skin barrier, etc.; UF vs. NF: Collagen fibril organization, etc. | \ | Wang et al., 2021 |
| Aging | Ginsenoside Rg3 | Human dermal fibroblast cell line | \ | \ | Jang et al., 2020 |
| Aging | Aqueous gel containing 6.1% of ingredients from marine and maritime origins | Human skin | Gene expression, cell survival and metabolism, inflammatory processes, etc. | \ | Hameury et al., 2018 |
| Aging | HA | Mice skin | \ | \ | Yun et al., 2017 |
| Photoaging | *M. thunbergi* | Mice skin | \ | \ | Uhm et al., 2010 |
| UVB-induced damage | Trehalose | Human skin | \ | Trehalose protects keratinocytes against UVB radiation by activating autophagy via regulating TIMP3 and ATG9A. | Li et al., 2022 |
| UVA-induced damage | Tectorignen | HaCaT cells | MAPK signaling pathway, cellular responses to UV, etc. | Tectorigenin can inhibit UVA induced oxidative stress injury and inflammatory response, and also inhibit the MAPK/JNK/AP-1 signalling pathway through targeting RAR-γ. | Dai et al., 2022 |
| UVA-induced damage | Thai silk sericins | Human primary epidermal melanocytes | Inflammatory response, negative regulation of apoptotic process, etc. | \ | Petpiroon et al., 2022 |
| UV-induced damage | CBD | Mice skin | Antioxidative activity, apoptosis, etc. | \ | Atalay et al., 2021 |
| UV-induced damage | CBD | Human epidermal keratinocytes | Antioxidant and anti-inflammatory activity, etc. | \ | Gęgotek et al., 2021 |
| UV-induced damage | QB | Reconstructed epidermis tridimensional cell lines | \ | \ | Camillo-Andrade et al., 2020 |
| UV-induced damage | Ascorbic acid and rutin | Human skin fibroblasts | DNA organization and expression, protein biosynthesis | \ | Gęgotek et al., 2020 |
| UV-induced damage | AAH | Human immortalized keratinocytes (Hacats) | Keratinization, carbohydrate catabolic and metabolic processes, etc. | \ | Zeng et al., 2019 |
| UV-induced damage | CBD | Human skin fibroblasts | Antioxidant, structural signalling catalytic, etc. | \ | Gęgotek et al., 2019 |
| AD | CLIC1 Knock-Down | Human A549 cells | \ | \ | Yin et al., 2021 |
| AD | Dupilumab | Human skin | \ | \ | Bangert et al., 2021 |
| AD | Dupilumab | Human skin tape [AD (female=4, male=22)] | Th2/Th17 pathway | \ | He et al., 2020 |
| AD | High humidity | Human skin | \ | Hyperosmotic stress upregulates CLCA2 via the p38/JNK-ATF2 signaling pathway. Increased CLCA2 expression stabilizes cell-to-cell connections. | Seltmann et al., 2018 |
| AD | IL-4, IL-13 treatment | Human skin [AD (n=21), HCs (n=12)] | \ | Interference of S100/A11 expression, by siRNA, inhibited induction of HBD-3 and FLG | Howell et al., 2008 |
| HS | Brodalumab subcutaneous injection | Human serum [HS (n=10)] | TNF response, neutrophil, general inflammatory responses | Higher expression of LCN2 in skin and IL-17A in serum correlates with a greater decrease of inflammatory cytokines with treatment | Navrazhina et al., 2022 |
| Vitiligo | GCs | Human urine [Treatment-effective (n = 42), Treatment-resistant (n = 16)] | Immune-related pathways, cellular growth, proliferation, etc. | \ | Qian et al., 2022 |
| Acne | Cryptotanshinone | Rats skin | Glycolysis/gluconeogenesis, galactose metabolism, etc. | \ | Zhu et al., 2021 |
| Tissue fibrosis | Blue light photobiomodulation | C57BL/6 mice dermal fibroblasts | \ | \ | Chang et al., 2020 |
| AGA | LLLT | Human dermal papilla tissues [AGA (n=4)] | Regulation of cellular transcription, translation,  protein biosynthesis, ECM structural constituent, basement membrane organization, etc. | \ | Panchaprateep et al., 2019 |
| CA | Hyperthermia | Human CA tissue | Participated in metabolic and catabolic processes, PI3K-AKT and Hippo signaling pathways, etc. | \ | Sun et al., 2019 |
| Leishmaniasis | Meglumine antimoniate | Human granulation tissue [Leishmaniasis (n=4)] | Neutrophil aggregation and degranulation, leukocyte migration during the inflammatory response, etc. | \ | Montoya et al., 2019 |
| Skin pruritus | Aprepitant | HaCaT keratinocytes cells | p53 pathway, etc. | \ | Kwatra et al., 2019 |
| Hidradenitis suppurativa | Brodalumab | Human serum | \ | IL-17RA inhibition by brodalumab decreases several pathogenic inflammatory axes in hidradenitis suppurativa. | Navrazhina et al., 2022 |
| Hypertrophic scars | GHRP6 | New Zealand male rabbits skin | Lipid metabolism, cytoskeleton arrangements, epidermal cells' differentiation, ECM dynamics | \ | Fernández-Mayola et al., 2018 |
| Precancerous skin condition actinic keratosis | IngMeb | Human skin cell lines | \ | IngMeb inhibit the activity of SLC25A20 in human cells | Parker et al., 2017 |
| RDEB | Losartan | Mice skin | \ | \ | Nyström et al., 2015 |
| Inflammatory skin diseases | rhTRX | Melanoma cell lines | Lipid metabolism-dependent pathway | \ | Han et al., 2014 |
| Skin ulceration | PRP dressings | Human skin [PRP (n=17), PPP (n=17)] | \ | Analysis of growth factors contained in PRP using antibody profiler arrays. | Roubelakis et al., 2014 |

(Abbreviation: CTCL: Cutaneous T-cell lymphomas; UV: Utraviolet; AD: Atopic dermatitis; HS: Hidradenitis suppurativa; AGA: Androgenetic alopecia; CA: Condyloma acuminata; RDEB: Recessive dystrophic epidermolysis bullosa; IACS: electron transport chain complex I inhibitor, IACS-010759; STN: Atorvastatin; CHI3LE: Chitinase 3-like 1; CTLA-4: Cytotoxic T-lymphocyte–associated antigen-4; PD-1: Programmed cell death protein-1; MAPKis: Mitogen-activated protein kinase inhibitors; BRAFis: B‐Raf proto‐oncogene, serine/threonine kinase inhibitors; MMTC: N-(4-methyl)phenyI-O-(4-methoxy)phenyl-thionocarbamate; NRF2: Nuclear factor erythroid 2-related factor 2; TIL: Tmor infiltrating lymphocyte; MEKis: MEK inhibitors; BSE-SFN: Broccoli sprout extract containing sulforaphane; UBE2N: Ubiquitin-conjugating enzyme E2 N; BETis: Bromodomain and extra‐terminal domain inhibitors; PA: Panduratin A; PDT: Photodynamic therapy; AlS2Pc: Aluminium di-sulphonated phthalocyanine; SAHA: Suberoylanilide hydroxamic acid; M8: Stilbene derivative-3,3',4,4',5,5'-hexahydroxystilbene; TPA: 12-O-tetradecanoyl-phorbol-13-acetate; ANBP: a tradition Chinese medicine composing of Agrimonia eupatoria L. (A, Xinzhi), Nelumbon ucifera Gaertn (N, Sanxiang), Boswellia carteri Biedw. (B, Chaowei), andTypha orientalis C. Presl (P, Xinzhi) with precise proportions; HSAM: Hypothermically stored amniotic membrane; OCT: Octenidine dihydrochloride; ALA: 5-aminolevulinic acid; ESWT: Extracorporeal shock wave therapy; CBD: Cannabidiol; ADSCs: Adipose-derived stem cells; HA: Hyaluronic acid; NO: Nitric oxide; GGA: Geranylgeranylacetone; JYG: Jueyin granules; TWS: Total withanolides; LLDT-8: (5R)-5-hydroxytriptolide; SMHF: S. macrophylla hexane fraction; SMEAF: S. macrophylla ethyl acetate fraction; n-3 PUFA: Omega-3 polyunsaturated fatty acid; QB: Quinoa bioester; AAH: Acetylated and amidated hexapeptide; CLIC1: Chloride intracellular channel 1; GCs: Glucocorticoids; LLLT: Low-level laser therapy; GHRP6: Growth hormone releasing peptide 6; IngMeb: Diterpenoid ester ingenol mebutate; rhTRX: Recombinant human thioredoxin; PRP: Platelet-rich plasma; ICIs: Immune checkpoint inhibitors; DMBA: 7, 12-dimethylbenz[a]anthracene; TS: Tape-stripped; EpSCs: Epidermal stem cells; MTX: Methotrexate; YSWH: YSW high-dose; YSWL: YSW low-dose; NC: UV free and corn oil; NF: UV free and fish oil; UC: UV and corn oil; UF: UV and fish oil; PPP: Platelet-poor plasma; mTOR: mechanistic target of rapamycin; ETM: Epithelial-mesenchymal transition; ROS: Reactive oxygen species; TCA: Trichloroacetic acid; ECM: Extracellular matrix; MMP: Matrix metalloproteinase; PPAR: Peroxisome proliferator-activated receptor; JAK-STAT: Janus kinase-signal transducer and activator of transcription; MHC: Major histocompatibility complex; TNF: Tumor necrosis factor; PI3K: Phosphatidylinositol-4,5-bisphosphate 3-kinase; AKT: Protein kinase B; NEK9: Never in mitosis gene A‐9; pCHK1: Phosphorylated checkpoint kinase 1; CDK: Cyclin‐dependent kinase; FRA1: Fos-like antigen 1; SOX10: Sex determining region Y-related HMG box 10; FAK: Focal adhesion kinase; PHB: Prohibitin; SIRT: Sirtuin; ERK1/2: Extracellular signal-regulated kinases 1 and 2 ; BD-2: β-Defensin 2; CLCA2: Chloride channel accessory 2; JNK: c-Jun N-terminal kinase; ATF2: Activating transcription factor 2; S100/A11: S100 calcium-binding protein A11; HBD-3: Human beta-defensin-3; FLG: Filaggrin; LCN2: Lipocalin-2)
