# Supplementary material for: Proteomics as a tool to improve novel insights into skin diseases: what we know and where we should be going
Source: Front Surg. 2022 Oct 21;9:1025557. doi: 10.3389/fsurg.2022.1025557 (PMC9633964; doi:10.3389/fsurg.2022.1025557)
Supplement: Supplementary file 5 [file Table5.docx]

**Supplemental table 5.** Mechanism of skin wound pathogenesis within proteomic analysis.

| **Type of disease** | **Sample** | **Highlighting mechanism** | **Depth mechanism** | **Ref.** |
| --- | --- | --- | --- | --- |
| Wound | Mice skin | \ | MAFB plays the vital role of regulating multiple genes implicated in wound healing, and is a potential therapeutic target in wound healing. | Inoue et al., 2022 |
| Chronic skin wound | Human [Chronic skin wounds (n=5), HCs (n=6)] | Cell adhesion, ECM organization, lysosomal components, gene expression, RNA processing | \ | Berberich et al., 2020 |
| Wound | Multiple strains of mice | \ | Trps1 acts at different stages of wound repair to reduce scarring and is essential for complete regenerative repair. | Mascharak et al., 2022 |
| Wound | Uninfected healing wounds (n=3); infected wounds (n=3) | \ | \ | van der Plas et al., 2021 |
| Wound | Human [Trauma wounds (n=11)] | \ | \ | Bekeschus et al., 2018 |
| Wound | Human [men (n=5), female (n=2)] | RNA gene silencing, cell activation, apoptosis, EGFR-related signaling pathways | \ | Gao et al., 2018 |
| Wound | Human | Cellular process, metabolic process, biological regulation,etc. | \ | Maarof et al., 2018 |
| Wound | Horse | Morphologically healthy, cell metabolism | \ | Harman et al., 2018 |
| Wound | Myofibroblasts | Protein metabolism, cell growth and/or maintenance, integrin family cell surface interactions, integrin cell surface interactions, etc | \ | Merjaneh et al., 2017 |
| Wound | Mice | \ | \ | Casado et al., 2014 |
| Wound | MRL/MpJ-Fas(lpr) mice | \ | \ | Caldwell et al., 2008 |
| Wound (in DM) | Human [Rapidly healing (RH, n = 17), Non-healing (NH, n = 11)] | Apoptosis, protease inhibitor activity, etc. | High SerpinB3 protein content was found to be a biomarker of successful healing in diabetic patients. | Fadini et al., 2014 |
| Wound (in T1DM) | Sprague-Dawley rats [T1DM (n=6), Controls (n=6)] | Regulation of the cytoskeleton, carbohydrate metabolism, protein folding | Annexin A2, may functioning as downstream of JNK, accelerated the rate of human skin fibroblast cell migration. | Wang et al., 2017 |
| Wound (in T2DM) | Human [T2DM (n=10), Burn (n=6)] | Cell organization and biogenesis, biological development, response to stimulation, etc. | \ | Krisp et al., 2013 |

(Abbreviation: DM: Diabetes mellitus; T1DM/T2DM: Type 1/Type 2 DM; HCs: Human controls; RH: Rapidly healing; NH: Non-healing; ECM: Extracellular matrix; JNK: c-Jun N-terminal kinase.)
