# Supplementary material for: Proteomics as a tool to improve novel insights into skin diseases: what we know and where we should be going
Source: Front Surg. 2022 Oct 21;9:1025557. doi: 10.3389/fsurg.2022.1025557 (PMC9633964; doi:10.3389/fsurg.2022.1025557)
Supplement: Supplementary file 6 [file Table6.docx]

**Supplemental table 6.** Mechanism of alopecia pathogenesis within proteomic analysis.

| **Type of disease** | **Sample** | **Highlighting mechanism** | **Depth mechanism** | **Ref.** |
| --- | --- | --- | --- | --- |
| FFA | Human [FFA (n=12), AA (n=18), HCs (n=8)] | \ | \ | Dubin et al., 2022 |
| Alopecia (in acute radiation) | C57/BL6 mice [X-rays (n=5), controls] | \ | \ | Nanashima et al., 2012 |
| Alopecia | Female human [19-81years] | \ | \ | Williams et al., 2021 |
| Alopecia | Human | Nucleotide excision repair, DNA mismatch repair, homologous recombination, MAPK pathway | \ | Haslam et al., 2021 |
| Alopecia | Dermal papilla cells | ECM-receptor interaction, focal adhesion, the TGF-β signaling pathway, cellular processes, etc. | \ | Zhang et al., 2016 |
| AGA | Human [male AGA (n=2)] | Protein folding, transfort and degradation, cell adhesion, cytoskeleton organization, etc. | \ | Moon et al., 2013 |
| AA | Human [AA (n = 35), HCs (n = 36), psoriasis (n = 19), AD (n = 49)] | Atherosclerosis signaling, immune pathways, cardiovascular pathway | \ | Glickman et al., 2021 |
| AA | Human [AA (n = 18), HCs (n = 8)] | \ | \ | Glickman et al., 2021 |
| AA | Human [AA (n=15), HCs (n=15)] | \ | \ | Ahn et al., 2011 |

(Abbreviation: FFA: Frontal fibrosing alopecia; AA: Alopecia areata; AGA: Androgenetic alopecia; HCs: Human controls; AD: Atopic dermatitis; MAPK: Mitogen-activated protein kinase; ECM: Extracellular matrix; TGF-β: Transforming growth factor-beta)
