# Supplementary material for: Proteomics as a tool to improve novel insights into skin diseases: what we know and where we should be going
Source: Front Surg. 2022 Oct 21;9:1025557. doi: 10.3389/fsurg.2022.1025557 (PMC9633964; doi:10.3389/fsurg.2022.1025557)
Supplement: Supplementary file 7 [file Table7.docx]

**Supplemental table 7.** Mechanism of SSC pathogenesis within proteomic analysis.

| **Type of disease** | **Sample** | **Highlighting mechanism** | **Depth mechanism** | **Ref.** |
| --- | --- | --- | --- | --- |
| SSc | Human [SSc (n=8), HCs (n=8)] | ECM-receptor interaction, PI3K-AKT signaling pathway, HIF-1 signaling pathway | The VEGF-a inhibitor bevacizumab treatment had the same effect on the hypoxic expression of α-SMA and CD31 | Mao et al., 2022 |
| SSc | Human [SSc (n=7)] | Platelet activation, ECM-receptor interaction, complement and coagulation cascades, antigen processing and presentation, leukocyte transendothelial migration | \ | Chairta et al., 2020 |
| lcSSc | Skin fibroblasts | Glycolysis, response to ROS, organization of cytoskeleton, cell homeostasis | \ | Corallo et al., 2016 |
| SSc | Human [SSc (n=3), HCs (n=3)] | \ | \ | Dumit et al., 2014 |
| SSc | Human [SSc (dcSSc) (n=19), Sc (lcSSc) (n=21), HCs (n=19)] | \ | \ | van Bon et al., 2014 |
| SSc | Huamn [SSc (n=12), HCs (n=12)] | \ | \ | Aden et al., 2008 |

(Abbreviation: SSc: Systemic sclerosis; lcSSc: Limited cutaneous SSc; HCs: Human controls; dcSSc: Diffuse cutaneous SSc; ECM: Extracellular matrix; PI3K: Phosphatidylinositol-4,5-bisphosphate 3-kinase; AKT: Protein kinase B; HIF-1: Hypoxia-inducible factor-1; ROS: Reactive oxygen species; VEGF-a: Vascular endothelial growth factor-a; α-SMA: α-smooth muscle actin)
