# Supplementary material for: Proteomics as a tool to improve novel insights into skin diseases: what we know and where we should be going
Source: Front Surg. 2022 Oct 21;9:1025557. doi: 10.3389/fsurg.2022.1025557 (PMC9633964; doi:10.3389/fsurg.2022.1025557)
Supplement: Supplementary file 8 [file Table8.docx]

**Supplemental table 8.** Mechanism of other skin diseases pathogenesis within proteomic analysis.

| **Type of disease** | **Sample** | **Highlighting mechanism** | **Depth mechanism** | **Ref.** |
| --- | --- | --- | --- | --- |
| Vulgaris acne | Human | Metabolic, immune, complement and coagulation cascades, apoptotic signaling pathway, etc. | \ | Quanico et al., 2017 |
| Vitiligo | Online data | **M**elanin biosynthetic process, inflammatory response, etc. | \ | Malhotra et al., 2019 |
| Vitiligo | Human [Vitiligo (n=22), HCs (n=14)] | \ | \ | Kim et al., 2011 |
| VEN | Human [VEN (n=67), inflammatory linear VEN (n=58)] | Th1 and Th2 cell differentiation, PPAR signaling pathway, etc. | \ | Yuan et al., 2022 |
| VEN | Human [VEN (n=5), HCs (n=5)] | Cornification, epithelial cell differentiation, neutrophil activation, oxidative phosphorylation, PPAR signalling pathway, etc. | \ | Yuan et al., 2021 |
| SLE | Human [SLE (n=310)] | \ | \ | Torell et al., 2019 |
| SLE | Human | \ | \ | Hudspeth et al., 2019 |
| SLE | Human [SLE + Skin lesions (n=21), SLE (n=18), HCs (n=22)] | \ | \ | Tan et al.,2015 |
| Skin sensitization | HaCaT cell line | \ | \ | Bailey et al., 2021 |
| Skin invasion by blood fluke larvae | Human | \ | \ | Hansell et al., 2020 |
| SGVHD | Human [SGVHD (n=10), No HCs (n=10)] | \ | Plasma concentrations of elafin were significantly higher at the onset of SGVHD, correlated with the eventual maximum grade of SGVHD. | Paczesny et al., 2010 |
| SCARs | Human [TOL (n = 9), MPE (n = 9), DRESS (n = 9), SJS/TEN (n = 8)] | Complement activation, immune response, acute inflammatory response, etc. | \ | Salinas-Jaramillo et al., 2021 |
| SARS‐CoV‐2 infection | Human [COVID-19 (n=5), HCs (n=6)] | Response to microorganisms, apoptosis, blood circulation, tissue remodeling, etc. | \ | Ma et al., 2022 |
| RDEB | Human [RDEB (n=6, age <1)] | \ | Altered TGF-β signaling in RDEB fibroblasts induces elevated Cbl levels. | Martínez-Martínez et al., 2021 |
| RDEB | Human [RDEB(n=4), HCs(n=3)] | ECM part, vesicle-mediated transport, cell adhesion, DNA-dependent DNA replication | \ | Küttner et al., 2014 |
| PV | Human [PV (n=7), HCs (n=5)] | \ | \ | Kalantari-Dehaghi et al., 2021 |
| Pressure ulcers | Human [Pressure ulcers (n=6), HCs (n=3)] | \ | \ | Taverna et al., 2011 |
| PPR | Human | MAPK signaling pathway, TNF signaling pathway | Stimulation of NLS explants with IL-1b induces a papulopustular-like transcriptomic and proteomic profile. | Harden et al., 2020 |
| PKDL | Human [MAC (n = 20), POLY (n = 20), CR (n = 12), HCs (n = 12)] | Inflammatory, tmmune response, transporter activity | \ | Jaiswal et al., 2020 |
| Photodamage | Human (n=6) | \ | \ | Voegeli et al., 2017 |
| Photodamage | Hos:HR-1 hairless mice | Cell death, survival, development, etc. | The regulation of the TCA cycle via the decrease of DLD is closely related to the malic acid and fumaric acid metabolites in the epidermis of the UVB-induced photodamage | Moon et al., 2015 |
| OcR | Human [OcR (n=14), HCs (n=10)] | \ | \ | Wladis et al., 2017 |
| Netherton syndrome | Human [Netherton syndrome (n=3), HCs (n=8)] | \ | \ | Bennett et al., 2012 |
| Mastocytosis | Human [HCs (n = 8), Psoriasis (n = 8), Mastocytosis (n = 4)] | \ | \ | Gschwandtner et al., 2016 |
| Localized scleroderma | Mice | Muscle contraction, acute-phase response, etc. | \ | Ma et al., 2022 |
| LFs following electrical injury | Human | Organismal death, cell death, etc. | \ | Lindford et al., 2021 |
| Leprosy | Human [LL (n=11), ENL (n=13)] | Blood coagulation, inflammatory response | \ | Silva et al., 2018 |
| Leishmaniasis | Human [Leishmaniasis (n=11), HCs (n=6)] | Cell death, cell adhesion, cell cycle, immune response | \ | da Silva Santos et al., 2015 |
| LDIR exposure | Keratinocytes | \ | LDIR irradiation suppresses ribosomal biogenesis via the upregulation of PP2A and downregulation of c-Myc and mTOR signaling. | Sekihara et al., 2018 |
| LA | Human [LA (n=10), HCs (n=10)] | Tissue development, epidermis development, anatomical structure development, etc. | \ | Cai et al., 2017 |
| KS | Human [KS (n=10), HCs (n=10)] | Energy metabolism, cytoskeleton, etc. | \ | Ong et al., 2010 |
| Keloid | Human | Cornification, keratinization, keratinocyte differentiation, etc. | \ | Liu et al., 2022 |
| Keloid | Human [Keloid (n=7), Melanoma (n=9)] | \ | \ | Barallobre-Barreiro et al., 2019 |
| Keloid | Human [Keloid (n=17)] | \ | \ | Lee et al., 2013 |
| Keloid | Human [Keloid (n = 8), HCs (n=4)] | Hemostasis, inflammation, proliferation, Toll-like receptor, ERK pathways, etc. | \ | Zhang et al., 2021 |
| Keloid | Human | Platelet aggregation, biological adhesion, blood vessel endothelial cell migration, etc. | \ | Yan et al., 2020 |
| Keloid | Human [Keloid (n=12), HCs (n=10)] | \ | Annexin A2 inhibited the proliferation of keloid fibroblasts | Kim et al., 2014 |
| Keloid | Human | \ | \ | Javad et al., 2012 |
| KD | Human [Febrile (n = 60), Nonfever (n = 60), KD (n = 60)] | \ | \ | Kuo et al., 2018 |
| Jet fuel induced damage | Human | Protein synthesis, folding, degradation, etc. | \ | Witzmann et al., 2005 |
| ILNEB | Human [ILNEB (n=3)] | \ | \ | He et al., 2018 |
| Ichthyoses | Mice [Lor knockout mice (n=7), Controls (n=8)] | \ | \ | Rice et al., 2016 |
| HZ | Human [HZ (n=40), HCs (n=40)] | \ | \ | Wang et al., 2020 |
| Hypertrophic scar intraepidermal blister | Human [Patients (n=3)] | \ | \ | Tan et al., 2015 |
| Hypertrophic scar | Human | Response to stimulus, developmental process, etc. | \ | Ma et al, 2014 |
| Hyperhidrosis | Primary sweat gland cells | \ | \ | Klaka et al., 2017 |
| Human hypertrophic scar | Human | \ | \ | Li et al., 2018 |
| HS | Human [HS, HCs] | \ | \ | Carmona-Rivera et al., 2022 |
| HS | GSE79150 | ROS pathway, adipogenesis, etc. | \ | Hoffman et al., 2018 |
| HHD | Human [HHD (n=3), HCs (n=3)] | PI3K‐AKT signaling pathway, focal adhesion, etc. | \ | Zhang et al., 2019 |
| Hair damage | Human | \ | \ | Sinclair et al., 2012 |
| GCMN | Human [GCMN (n=10), HCs (n=10)] | Posttranslational modification, cytoskeleton, neurotrophin signaling pathway, glycolysis/gluconeogenesis | \ | Kim et al., 2012 |
| Fungal allergy | Human [Non-atopic (n=6), Atopic (n=22)] | \ | \ | Dey et al., 2016 |
| Fibroblast senescence | Human skin fibroblast cell line | RNA processing, mitotic nuclear division, etc. | \ | Meng et al., 2018 |
| Familial Hidradenitis Suppurativa | NCBI Gene Expression Omnibus(skin, blood) | Apoptosis, cell proliferation offibroblasts, cell proliferation offibroblasts | \ | Frew et al., 2019 |
| Early-stage cutaneous mycosis fungoides | Human [Early-stage (n=4), Advanced‐stage (n=10)] | Bicarbonate transport, transcription, etc. | \ | Leng et al., 2022 |
| Epidermal barrier dysfunction | Human | Keratinocyte differentiation, epidermal cell differentiation, etc. | Epidermal loss of ceramide synthase 4 first disturbs epidermal lipid metabolism and adult epidermal barrier function. | Peters et al., 2020 |
| Dry skin | Human [Postmenopausal women (n=28), Young women (n=30)] | \ | \ | Delattre et al., 2012 |
| Drug-induced TEN | Human | \ | \ | Paquet et al., 2012 |
| DM, SLE | Human [DM (n=25), SLE (n=20), HCs (n=15)] | \ | \ | Nakamura et al., 2016 |
| DM | Human | \ | \ | Salajegheh et al., 2010 |
| DFU | Human [DFU (n=18), HCs (n=3)] | \ | \ | Hoke et al., 2016 |
| Dandruff | Human [Dandruff (n=9), No dandruff (n=10)] | Epidermal differentiation and barrier function, antioxidant defense | \ | Cavusoglu et al., 2016 |
| CSU | Human [ASST-positive (n=3), ASST-negative (n=3)] | \ | ASST results and serum clusterin levels can predict 92.7% of CSU patients whose urticaria would be refractory to antihistamines. | Kim et al., 2016 |
| Corneocyte desquamation | Human (n=9) | \ | \ | Lin et al., 2012 |
| CHE | Human [CHE (n=6), HCs (n=6)] | \ | \ | Molin et al., 2015 |
| Bowen disease | Human [CSCC (n = 5), Bowen disease (n = 5), HCs (n = 5)] | Nucleobase-containing compound catabolic process, cytokine-mediated signaling pathway, granulocyte activation, etc. | TNC, FSCN1, SERPINB1, ACTN1, RAB31, COL3A1, COL1A1, CD36 were significantly associated with the mechanisms of invasion and metastasis in Bowen's disease. | Biao et al., 2022 |
| BP | Human [BP (n=8)] | Innate immune response, neutrophil degranulation, etc. | \ | Solimani et al., 2021 |
| BP | Human [BP (n=16), HCs (n=8)] | Innate immune response, proteolysis, cytoskeleton organization, inflammatory response, etc. | \ | Fang et al., 2018 |
| ARCI | Human [ARCI (n=10), HCs (n=7)] | \ | \ | Karim et al., 2019 |
| AK | Human [AK (n=5)] | Proteoglycan syndecan-mediated signaling, PI3K signaling, mTOR signaling, IL-5 signaling, EGF signaling pathways | \ | Azimi et al., 2019 |
| AE | Epidermal keratinocytes | Inflammatory, proteolytic, cytoskeletal dysregulation | \ | Elias et al., 2017 |
| AE | Human [AE (n=18), HCs (n=16)] | \ | \ | Holm et al., 2014 |
| Acne vulgari | Human [Acne vulgari (n=20), HCs (n=18)] | Response or defense to a bacterium, etc. | \ | Bek-Thomsen et al., 2014 |
| Acne | Human | \ | Following 3 days in the presence of 300 lg/mL SMFE, the RNA encoding K75 was upregulated by 15-fold. | Fontao et al., 2020 |
| Aging | Human primary skin fibroblasts | Collagen catabolic, endosomal vesicle fusion, etc. | \ | Tsitsipatis et al., 2022 |
| Aging | Fibroblasts | \ | \ | Mao et al., 2022 |
| Aging | Mice skin | \ | TMPRSS11a is overexpressed in aged skin. | Fernandez et al., 2022 |
| Aging | Normal human epidermal keratinocytes | \ | Glyoxal can affect keratinocyte functions and act as a driver of human skin aging | Halkoum et al., 2022 |
| ACC | Human | RNA-posttranscriptional modification, cell cycle, etc. | \ | Marneros et al., 2013 |

(Abbreviation: HS: Hidradenitis suppurativa; SCARs: Severe cutaneous adverse reactions; VEN: Verrucous epidermal naevi; PV: Pemphigus vulgaris; RDEB: Recessive dystrophic epidermolysis bullosa; LFs: Lichtenberg Figures; BP: Bullous pemphigoid; HZ: Herpes zoster; PPR: Papulopustular rosacea; PKDL: Post Kala Azar Dermal Leishmaniasis; HHD: Hailey-Hailey disease; SLE: Systemic lupus erythematosus; AK: Actinic keratoses; ARCI: Autosomal Recessive Congenital Ichthyosis; LDIR: Low-dose ionizing radiation; ILNEB: Interstitial lung disease, nephrotic syndrome and epidermolysis bullosa; KD: Kawasaki disease; OcR: Ocular rosacea; AE: Atopic eczema; LA: Lichen amyloidosis; CSU: Chronic spontaneous urticaria; DFU: Diabetic foot ulcers; CHE: Chronic hand eczema; ACC: Aplasia cutis congenita; GCMN: Giant congenital melanocytic nevus; TEN: Toxic epidermal necrolysis; SGVHD: Skin graft-versus-host disease; KS: Keloid scar; HCs: Human controls; TOL: drug-tolerant controls; MPE: maculopapular exanthema; DRESS: Drug reaction with eosinophilia and systemic symptoms; SJS: Stevens−Johnson syndrome; MAC: Macular PKDL; POLY: Polymorphic PKDL; CR: Cured PKDL; LL: Lepromatous leprosy; ENL: Erythema nodosun leprosum; DM: Dermatomyositis; ASST: Autologous serum skin test; PPAR: Peroxisome proliferator-activated receptor; MAPK: Mitogen-activated protein kinase; TNF: Tumor necrosis factor; PI3K: Phosphatidylinositol-4,5-bisphosphate 3-kinase; AKT: Protein kinase B; mTOR: mechanistic target of rapamycin; EGF: Epidermal growth factor; ECM: Extracellular matrix; ROS: Reactive oxygen species; TGF-β: Transforming growth factor-beta; Cbl: Casitas B lymphoma; SMFE: Silybum marianum fruit extract; K75: Keratin 75; NLS: ; IL-1b: Interleukin-1beta; PP2A: Protein phosphatase 2A; TCA: Tricarboxylic acid; DLD: Dihydrolipoyl dehydrogenase; UVB: Ultraviolet B; TMPRSS11a: Transmembrane protease serine 11a)
