# Supplementary material for: Proteomics as a tool to improve novel insights into skin diseases: what we know and where we should be going
Source: Front Surg. 2022 Oct 21;9:1025557. doi: 10.3389/fsurg.2022.1025557 (PMC9633964; doi:10.3389/fsurg.2022.1025557)
Supplement: Supplementary file 9 [file Table9.docx]

**Supplemental table 9.** Reseaches in pathogenic microorganisms used proteomics.

| **Pathogenic microorganisms.** | **Main type of disease** | **Ref.** |
| --- | --- | --- |
| Acanthamoeba | [Cutaneous amebiasis](D:\\Program Files (x86)\\Youdao\\Dict\\8.10.5.0\\resultui\\html\\index.html" \o "D:\\Program Files (x86)\\Youdao\\Dict\\8.10.5.0\\resultui\\html\\index.html) | Deng et al., 2015 |
| *Bacillus anthracis* | Anthrax | Ghosh et al., 2014 |
| *Bacillus oleronius* | Papulopustular rosacea | Maher et al., 2018 |
| Chikungunya virus | Maculopapular rash | Issac et al., 2014 |
| CHIKV, ZIKV | \ | Wichit et al., 2019 |
| *Corynebacterium jeikeium* | Nosocomial infections | Hansmeier et al., 2007 |
| *Ctenocephalides felis* | Papular urticaria | Sabogal et al., 2019 |
| *Cutibacterium acnes* | [Acne](D:\\Program Files (x86)\\Youdao\\Dict\\8.10.5.0\\resultui\\html\\index.html" \o "D:\\Program Files (x86)\\Youdao\\Dict\\8.10.5.0\\resultui\\html\\index.html) | Borrel et al., 2019 |
| *Helicobacter pylori* | Rosacea, Chronic urticaria | Mini et al., 2005 |
| Herpes simplex virus | Herpes | Hasan et al., 2019 |
| Herpes viruses | Many types | Cheerathodi et al., 2020 |
| Herpes viruses | Herpes | Suk et al., 2015 |
| House dust mites | Cutaneous allergic diseases | Sookrung et al., 2018 |
| House dust mites | Cutaneous allergic diseases | An et al., 2013 |
| Itch mite Sarcoptes scabiei | Pruritic skin disease scabies | Morgan et al., 2016 |
| *L. amazonensis* | Cutaneous leishmaniasis | Oliveira et al., 2020 |
| *Leishmania* | Cutaneous leishmaniasis | Pissarra et al., 2022 |
| *Leishmania* | Cutaneous leishmaniasis | Shermeh et al., 2021 |
| *Leishmania* | Cutaneous leishmaniasis | Rodríguez-Vega et al., 2021 |
| *Leishmania* | Cutaneous leishmaniasis | Tasbihi et al., 2020 |
| *Leishmania* | Cutaneous leishmaniasis | Negrão et al., 2019 |
| *Leishmania* | Cutaneous leishmaniasis | Norris-Mullins et al., 2018 |
| *Leishmania* | Cutaneous leishmaniasis | Valdivia et al., 2015 |
| *Leishmania* | Cutaneous leishmaniasis | Duarte et al., 2015 |
| *Leishmania* | Cutaneous leishmaniasis | Teixeira et al., 2015 |
| *Leishmania* | Cutaneous leishmaniasis | Hassani et al., 2011 |
| *Leishmania (Viannia) braziliensis* | Cutaneous leishmaniasis | Esteves et al., 2022 |
| *Leishmania braziliensis* | Cutaneous leishmaniasis | Garcia et al., 2021 |
| *Leishmania braziliensis* | Cutaneous leishmaniasis | E Silva et al., 2020 |
| *Leishmania braziliensis* | Cutaneous leishmaniasis | E Silva et al., 2016 |
| *Leishmania braziliensis* | Cutaneous leishmaniasis | Alves-Ferreira et al., 2015 |
| *Leishmania braziliensis* | Cutaneous leishmaniasis | Cuervo et al., 2007 |
| *Leishmania donovani* | Post kala-azar dermal leishmaniasis | Routaray et al., 2022 |
| *Leishmania donovani* | Cutaneous leishmaniasis | Ravooru et al., 2014 |
| *Leishmania donovani* | Cutaneous leishmaniasis | Tsigankovt al., 2013 |
| *Leishmania infantum* | Cutaneous leishmaniasis | Timm et al., 2017 |
| *Leishmania major* | Cutaneous leishmaniasis | Jha et al., 2020 |
| *Leishmania major* | Cutaneous leishmaniasis | Amiri-Dashatan et al., 2020 |
| *Leishmania major* | Cutaneous leishmaniasis | Pawar et al., 2017 |
| *Leishmania major* | Cutaneous leishmaniasis | Zarean et al., 2015 |
| *Leishmania major* | Cutaneous leishmaniasis | Pawar et al., 2014 |
| *Leishmania major, Leishmania infantum* | Cutaneous leishmaniasis | John et al., 2012 |
| *Leishmania mexicana, Leishmania infantum* | Cutaneous leishmaniasis | Lynn et al., 2013 |
| *Leishmania pifanoi* | Cutaneous leishmaniasis | Alcolea et al., 2016 |
| *Leishmania* spp. | Cutaneous leishmaniasis | Negrão et al., 2019 |
| *Leishmania* spp. protozoa | Cutaneous leishmaniasis | Marshall et al., 2018 |
| *Leishmania tropica* | Cutaneous leishmaniasis | Mhaidi et al., 2021 |
| *Leishmania tropica* | Cutaneous leishmaniasis | Hajjaran et al., 2012 |
| *Leishmania tropica* | Cutaneous leishmaniasis | Mojtahedi et al., 2008 |
| *Leishmania Tropica* and Leishmania Major | Cutaneous leishmaniasis | Ashrafmansouri et al., 2019 |
| *Leishmania Tropica*, Leishmania Major | Cutaneous leishmaniasis | Amiri-Dashatan et al., 2021 |
| *Leishmania Tropica*, Leishmania Major | Cutaneous leishmaniasis | Ashrafmansouri et al., 2020 |
| *Leishmania Tropica*, Leishmania Major | Cutaneous leishmaniasis | Hajjaran et al., 2015 |
| *Leishmania Viannia* subgenus | Cutaneous leishmaniasis | Walker et al., 2006 |
| *Malassezia sympodialis* | Herpes | Corzo-León et al., 2020 |
| *Malassezia sympodialis* | Atopic eczema/dermatitis, Dandruff | Giotiet al., 2013 |
| *Megalopyge lanata, Podalia orsilochus* | Burning pain, Edema, Erythema | Sánchez et al., 2019 |
| Merkel cell polyomavirus | Merkel cell carcinoma | Stakaitytė et al., 2018 |
| Methicillin-resistant *Staphylococcus aureus* | AD | Chou et al., 2019 |
| MRSA | MRSA skin infection | Thangamani et al., 2015 |
| *Mycobacterium ulcerans* | Buruli ulcer | Dreyer et al., 2015 |
| *Mycobacterium ulcerans* | Buruli ulcer | Tafelmeyer et al., 2008 |
| *Propionibacterium acnes* | Acne | Jeon et al., 2017 |
| *Propionibacterium acnes* | Acne vulgaris | Yu et al., 2016 |
| *Propionibacterium acnes* | Acne | Dekio et al., 2015 |
| *Propionibacterium acnes* | Acne | Dekio et al., 2013 |
| *Propionibacterium acnes* | Acne | Holland et al., 2010 |
| *Pseudomonas* *aeruginosa* | \ | Latendorf et al., 2019 |
| *Sporothrix schenckii* | Sporotrichosis | Zhang et al., 2012 |
| *Staphylococcus aureu*, Bacillus thuringiensis, Klebsiella oxytoca | Wound infection | García-Pérez et al., 2018 |
| *Staphylococcus aureus* | Skin infection | Tu et al., 2021 |
| *Staphylococcus aureus* | Skin infection | Nagel et al., 2018 |
| *Staphylococcus aureus* | Skin infection | Yang et al., 2018 |
| *Staphylococcus aureus* | Skin infection | Das et al., 2016 |
| *Staphylococcus aureus* | Skin infection | Strobel et al., 2016 |
| *Staphylococcus aureus* | Skin infection | Enany et al., 2014 |
| *Staphylococcus aureus* | Skin infection | François et al., 2014 |
| *Staphylococcus lugdunensis* | Skin infection | Aubourg et al., 2022 |
| *Trichophyton rubrum* | Skin fungal infections | Corzo-León et al., 2019 |
| *Trichophyton rubrum* | Skin infection | Leng et al., 2008 |
| Varicella-zoster virus | Chickenpox and shingles | Lenac Roviš et al., 2013 |

(Abbreviation: AD: Atopic dermatitis; CHIKV: Chikungunya virus; ZIKV: Zika virus; MRSA: Methicillin-resistant Staphylococcus aureus)
