# Supplementary material for: Proteomics as a tool to improve novel insights into skin diseases: what we know and where we should be going
Source: Front Surg. 2022 Oct 21;9:1025557. doi: 10.3389/fsurg.2022.1025557 (PMC9633964; doi:10.3389/fsurg.2022.1025557)
Supplement: Supplementary file 10 [file Table10.docx]

| Type of disease | Ref. |
| --- | --- |
| Wound | Kur-Piotrowska et al., 2018 |
| Wound | Caldwell et al., 2008 |
| Skin tumor | Oh et al., 2007 |
| Skin tumor | Shen et al., 2007 |
| Psoriatic inflammation | Lagus et al., 2019 |
| Pressure Ulcer | Alexandrushkina et al., 2020 |
| Melanoma | Szadai et al., 2021 |
| Melanoma | Joyce et al., 2017 |
| Melanoma | Schütz et al., 2016 |
| Melanoma | Jiang et al., 2016 |
| Melanoma | Lazova et al., 2012 |
| Lymphomas | Bonnekoh et al., 2008 |
| Deep second-degree burn | Zhang et al., 2016 |
| AD | Shin et al., 2020 |
| \ | Cowen et al., 2021 |
| \ | Sawada et al., 2021 |
| \ | Tomalin et al., 2020 |
| \ | Rezaei-Tavirani et al., 2019 |
| \ | Desai et al., 2016 |
| \ | Neumann et al., 2015 |
| \ | Tilton et al., 2015 |
| \ | Pan et al., 2014 |
| \ | Tholen et al., 2013 |
| \ | Xie et al., 2009 |

**Supplemental t**able 10. Other researches of skin diseases within proteomic analysis.

(Abbreviation: AD: Atopic dermatitis)
