# Supplementary figures and images for: Proteomics as a tool to improve novel insights into skin diseases: what we know and where we should be going
Source: Front Surg. 2022 Oct 21;9:1025557. doi: 10.3389/fsurg.2022.1025557 (PMC9633964; doi:10.3389/fsurg.2022.1025557)

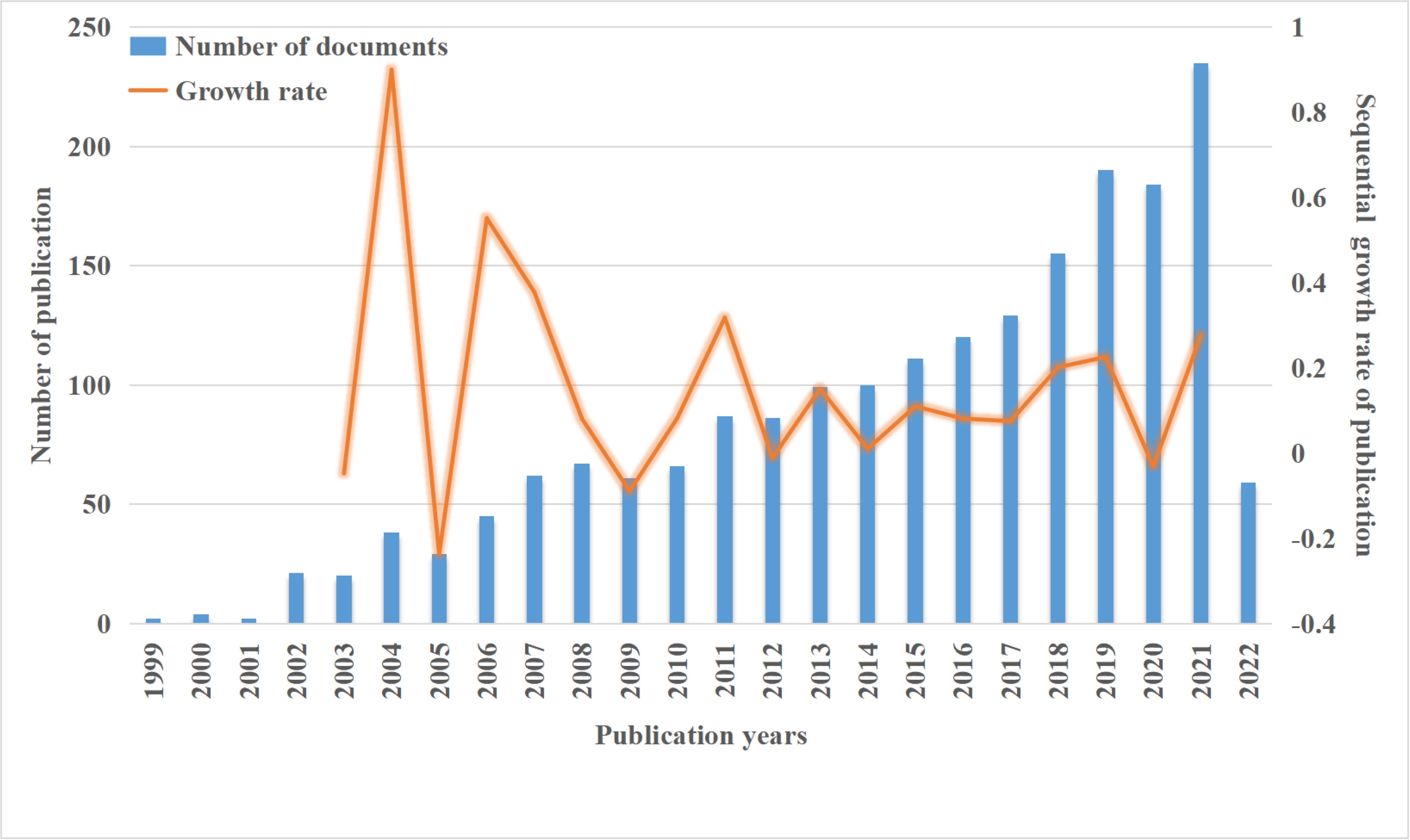

Supplement: Supplementary file 11 [file Image1.jpeg]

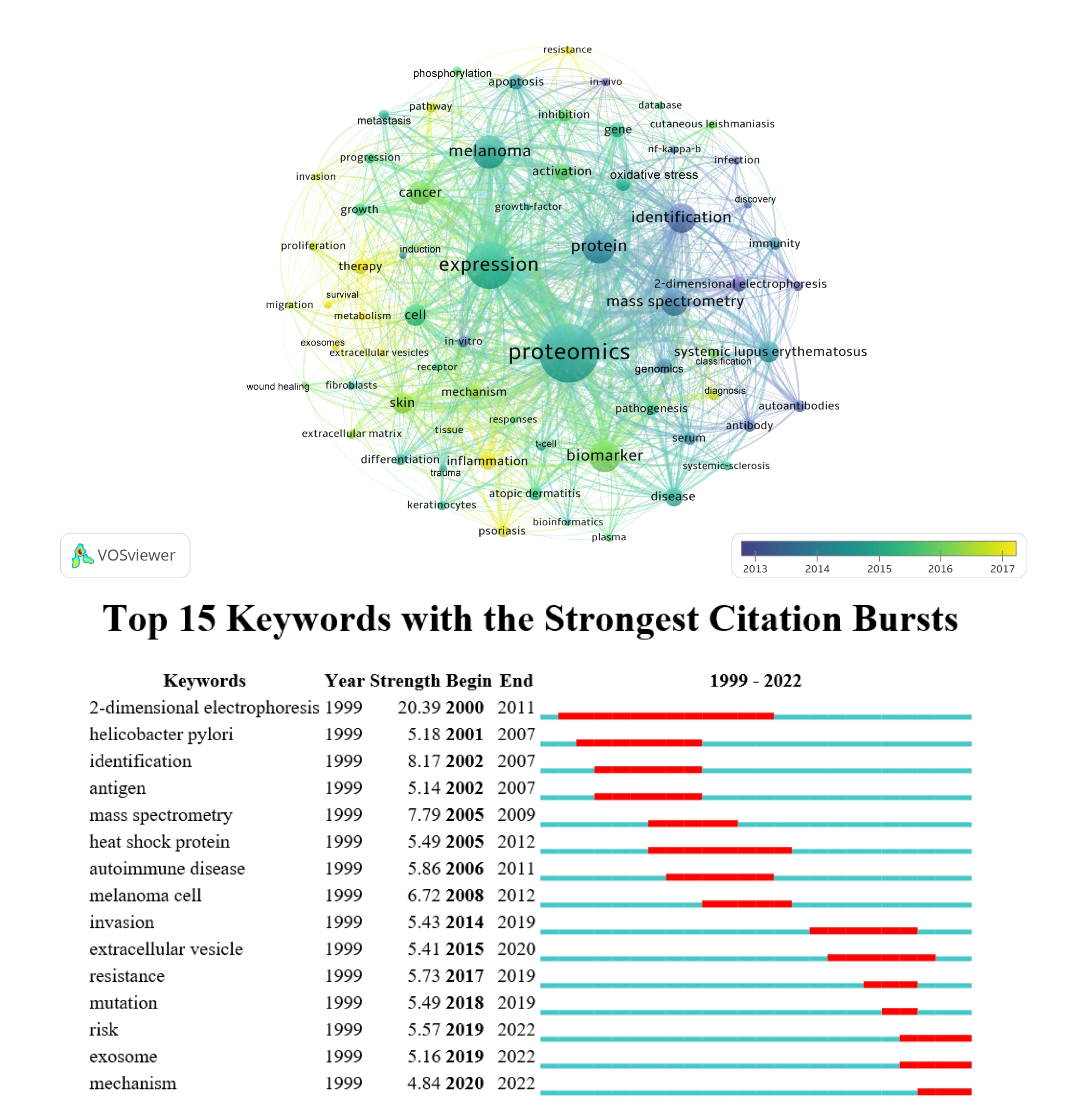

Supplement: Supplementary file 12 [file Image2.jpeg]
